# Fungal suppression by bacterial metabolite-activated human intestinal γδT-cells is disrupted in Crohn’s disease

**DOI:** 10.1371/journal.pbio.3004080

**Published:** 2026-11-02

**Authors:** Liya Mathew, Sean L. Carlson, Michael Savage, Claire Pardieu, Megan B. O’Brien, Ellie Crawley, Paul Harrow, Ann-Kristin Kaune, Edward Devlin, Emily L. Priest, Marilena Crescente, Kimberly Martinod, James Boot, SoFong Cam Ngan, Marco Gasparetto, Klaartje Kok, James O. Lindsay, Jonathan P. Richardson, Julian R. Naglik, Andrew J. Stagg, Matthias Eberl, Siu Kwan Sze, Carol A. Munro, Neil E. McCarthy

**Affiliations:** 1 Centre for Immunobiology and Infection, The Blizard Institute, Barts and The London School of Medicine and Dentistry, Queen Mary University of London, London, United Kingdom; 2 Institute of Medical Sciences, School of Medicine, Medical Sciences and Nutrition, University of Aberdeen, Aberdeen, United Kingdom; 3 Centre for Host-Microbiome Interactions, Faculty of Dentistry, Oral and Craniofacial Sciences, King’s College London, London, United Kingdom; 4 School of Pharmacy, University of Reading, Reading, United Kingdom; 5 Aab Cardiovascular Research Institute, Department of Medicine, University of Rochester School of Medicine and Dentistry, Rochester, New York, United States of America; 6 Faculty of Applied Health Sciences, Brock University, St. Catharines, Ontario, Canada; 7 Department of Paediatric Gastroenterology, The Royal London Children’s Hospital, Barts Health NHS Trust, London, United Kingdom; 8 Department of Gastroenterology, Barts Health NHS Trust, London, United Kingdom; 9 Division of Infection and Immunity, School of Medicine, and Systems Immunity Research Institute, Cardiff University, Cardiff, United Kingdom; University of Pennsylvania, UNITED STATES OF AMERICA

## Abstract

Gut symbionts condition mucosal immunity to resist infection by enteropathogens, but the specific microbes and mechanisms involved differ significantly between host species. In higher primates, bacterial metabolite HMB-PP is sensed by a specialized population of Vγ9Vδ2+T-cells, which we now report can potently suppress growth of endogenous fungi in human intestinal organ cultures. In healthy intestine, HMB-PP-stimulated Vδ2+ T-cells restricted outgrowth of keystone fungus *Candida albicans* via a mechanism that required IL-22. In Crohn’s disease (CD), Vδ2+ T-cells were poorly responsive to HMB-PP and permitted outgrowth of *C. albicans* strains that formed toxin-producing filaments, triggered neutrophil extracellular traps, and induced macrophage IL-1β release *ex vivo*. Genomic and proteomic analysis of the *Candida* isolates suggested increased tissue adhesion of CD-derived strains, which rapidly invaded the gut barrier in an ‘intestine-on-a-chip’ model. These data reveal that bacterial activation of Vδ2+ T-cells suppresses fungal pathobionts in human gut via an IL-22-dependent mechanism that is dysregulated in CD.

## Abstract 2

Fungi are common members of the gut microbiome, but inflammation can tip them toward harmful forms. This study shows that bacterial signals activate gut γδ T cells to suppress fungi and limit invasive strains and that this defense is disrupted in Crohn’s disease.

## Introduction

Commensal microbes condition mucosal immunity to protect against pathogen invasion and limit infection-related tissue damage [1–3], but the specific species and mechanisms involved differ between host animals and the diverse communities that colonize them. Commensal metabolite induction of host responses has been shown to limit colitis in mice infected with *Helicobacter hepaticus* [4], and modify the course of *Salmonella typhimurium* infection in both rodents [5] and nematodes [6], whereas disruption of these mechanisms can increase risk of mucosal inflammation [7]. However, several major enteropathogens of primates including *Norovirus, Shigella* bacteria, and *Candida albicans* fungus are absent or avirulent in small animal models, hence the commensal-driven mechanisms that naturally limit these infections remain poorly understood.

*C. albicans* routinely colonizes human gut as a commensal yeast [8], but changes in the host environment can stimulate the formation of tissue-invasive hyphal filaments which secrete candidalysin (CLYS) toxin and damage epithelial barriers. *C. albicans* invasion of gut tissue and damage to resident leukocytes is now thought to play a major role in inflammatory bowel disease (IBD) [9], and can progress to disseminated infection with >50% mortality in immunocompromised patients, hence the World Health Organization recently identified this species as a critical priority pathogen [10]. Although *C. albicans* is known to be a potent inducer of mucosal and systemic Th17 immunity in humans [11], the key cell types and cytokines that mediate fungal control vary significantly by body site, epithelial structure, and local conditions [12,13]. Current knowledge of the mechanisms that restrict *C. albicans* invasion of the human gut are limited, and since this species does not normally colonize mouse intestine [14], it remains unclear how the fungus impacts mucosal immunity and inflammation within a natural host environment.

Mucosal barrier sites are highly enriched in unconventional lymphocytes including γδ T-cells [15], which exhibit divergent expression of antifungal cytokine IL-17 in mice versus humans [16]. Rodents also completely lack the bacteria-responsive Vδ2+ population [17], which in primates is one of the most common T-cell lineages with defined antigen specificity [15]. In the current report, we observed that Vδ2+ T-cell activation by bacterial metabolite (*E*)-4-Hydroxy-3-methyl-but-2-enyl pyrophosphate (HMB-PP) can suppress a range of fungal pathobionts in human gut tissue via an IL-22-dependent mechanism that is dysregulated in Crohn’s disease (CD). *C. albicans* strains derived from CD gut tissue cultures readily formed toxin-producing filaments, triggered release of neutrophil extracellular traps, and induced macrophage IL-1β release *ex vivo*. Phenotypic, genomic, and proteomic analysis of the *C. albicans* isolates suggested increased tissue adhesion and invasion of CD-derived strains, which rapidly translocated the epithelial barrier in a human ‘gut-on-a-chip’ model subject to physiological forces of liquid flow and peristaltic stretch. Together, these data uncover a novel role for Vδ2+ T-cells in restricting the growth of human gut-adapted *C. albicans* strains with virulent features that have been implicated in IBD.

## Results

### Bacterial activation of human intestinal Vδ2+ T-cells restricts growth of fungal pathobionts

Vγ9Vδ2+ (Vδ2+) T-cells are ‘unconventional’ lymphocytes that uniquely respond to BTN3A-mediated sensing of the microbial metabolite HMB-PP [18,19], which is produced by a wide range of human commensal bacteria but not fungi [18]. We previously reported that Vδ2+ T-cells can promote IL-22 expression by conventional gut T-cells and induce calprotectin release in mucosal biopsy cultures [20]. To explore how this impacts gut barrier defense, we established human intestinal organ cultures with intact epithelium, which is the primary target of IL-22 signaling [21]. Strikingly, we observed that colonic tissue cultured under standard conditions displayed frequent outgrowth of endogenous fungi, but this did not occur when cultures were supplemented with HMB-PP to selectively activate Vδ2+ T-cells (Fig 1A and 1B). Indeed, HMB-PP activation of mucosal Vδ2+ T cells restricted growth of a variety of fungal species isolated from human intestinal tissue, including a range of thermotolerant molds and yeasts (Fig 1C–1F), the most prevalent of which was *C. albicans* (11 of 17 isolates; S1 Table). These data are consistent with reports that *C. albicans* dominates the subset of fungi that can colonize the intestinal mucus layer, whereas a more diverse range of transient species are detected in the gut lumen [22].

**Fig 1:**
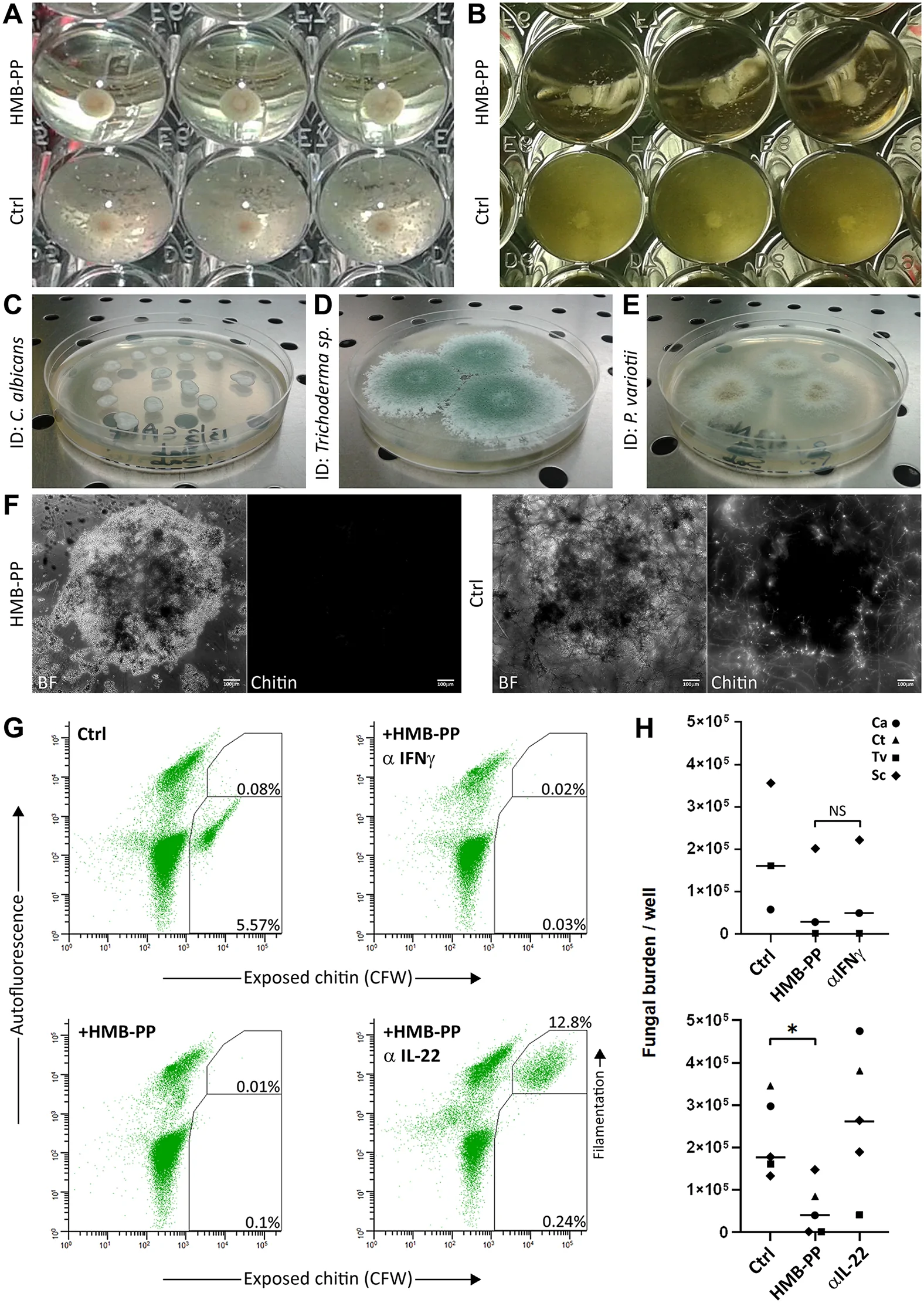
Bacterial activation of human intestinal Vδ2+ T-cells suppresses fungal growth. Human colonic organ cultures were stimulated or not with bacterial phosphoantigen HMB-PP (10nM) for 3 days at 37 °C and 5% CO_2_, which restricted outgrowth of endogenous yeasts **(A)** and molds **(B)**, including polymorphic *C. albicans* **(A, C)**, and filamentous species *Trichoderma viride* **(B, D)**, and *Paecilomyces variotii* **(E, F)**. Images show round-bottom chambers of a 96-well plate containing gut tissue-derived cells after 3 days either photographed from below **(A and B)**, cultured on Sabouraud agar **(C, D, E)**, or stained with calcofluor white prior to wide-field imaging at x10 magnification **(F)** to visualize cellular structures (brightfield; BF) and exposed chitin (scale bars indicate 100 μM). Supplementation of cultures with neutralising anti-cytokine antibodies and subsequent analysis by flow-cytometry revealed a key role for IL-22 in Vδ2+ T cell-mediated fungal control, whereas IFNγ neutralisation or an isotype-matched control had minimal effect (**G**; *T. viride* species shown; y-axis autofluorescence reflects increased mold filamentation). Quantification of fungal burden in gut tissue cultures from n = 5 control donors confirmed that HMB-PP induced significant restriction of endogenous fungal growth (p = 0.0178 by ANOVA), but not when IL-22 was neutralised **(H)**. Symbols indicate mucosal tissue-derived isolates of *C. albicans* (**Ca**)*, C. tropicalis* (**Ct**)*, T. viride* mold (**Tv**), or spiked-in *C. albicans* reference strain SC5314 (**Sc**). The data underlying this Figure can be found in https://doi.org/10.5281/zenodo.23064172.

We previously showed that Vδ2+ T-cell activation in human mucosal biopsies upregulates colonic CD4+ T-cell expression of IL-22 and IFNγ, each of which mediates a distinct phase of antifungal defense [23]. We therefore used blocking antibodies to test if either of these cytokines was critical for fungal suppression in HMB-PP-treated mucosal tissue cultures. While neutralization of IFNγ had little impact on fungal suppression in these assays, blockade of IL-22 severely disrupted suppression of intestinal Candida isolates, *C. albicans* reference strain SC5314, or *Trichoderma* mold (Fig 1G and 1H). These data indicate that HMB-PP is not directly anti-fungal and instead triggers Vδ2 T-cell induction of IL-22 and calprotectin as reported previously [20], thereby mediating colonization resistance against opportunistic pathogens [24].

### Vδ2+ T cell-mediated fungal suppression is impaired in patients with Crohn’s disease

IBD is strongly associated with genetic variants that impact major fungal receptors and pathways (including Dectin-1/CLEC7A, NOD2, and CARD9) [25] as well as IL-22 signaling in the gut epithelium [26–28]. We previously reported that Vδ2+ T-cells can act as potent inducers of IL-22 in blood and gut lymphocytes from healthy individuals [20], but it was unclear if this mechanism remains intact and able to suppress intestinal fungi in IBD. Indeed, unlike gut biopsies from control donors, mucosal tissue from CD patients contained only trace numbers of Vδ2+ T-cells which did not expand significantly upon HMB-PP treatment (Fig 2A and 2B) and failed to limit the growth of endogenous fungal species or protect cultures spiked with highly pathogenic *C. albicans* reference strain SC5314 (Fig 2C and 2D). To test if this defect was due only to low cell numbers or rather functional changes in disease, we next assessed whether Vδ2+ T-cells from CD patients can be stimulated to adopt an IL-22-inducing profile. To obtain sufficient cells for these assays we performed magnetic enrichment of peripheral blood Vδ2+ T-cells, which are chiefly integrin β7+ ‘gut-tropic’ cells in patients with CD [29], and then tested their ability to stimulate cytokine expression in sort-purified naïve CD4+ T-cell responders (S1 Fig). While circulating Vδ2+ T-cells from CD patients were able to acquire a co-stimulatory profile upon HMB-PP exposure (Fig 2E), these cells displayed significantly impaired antigen-presenting phenotype in patients with active CD compared with inactive disease (Fig 2F), resulting in poor IL-22 induction in responder CD4+ T-cells (Fig 2G and 2H). Taken together, these data reinforce that HMB-PP lacks direct anti-fungal activity and instead depends on Vδ2+ T-cell activation and IL-22 induction to limit fungal outgrowth, which in CD patients is disrupted by both numerical and functional deficits in the Vδ2+ T-cell compartment. Our findings are also consistent with murine data showing that IL-22 can restrain *C. albicans*-induced release of pro-inflammatory IL-1β [30], which correlates with colitis severity in human patients [9], whereas actively inflamed mucosal tissue is instead depleted of IL-22-producing T-cells [31].

**Fig 2:**
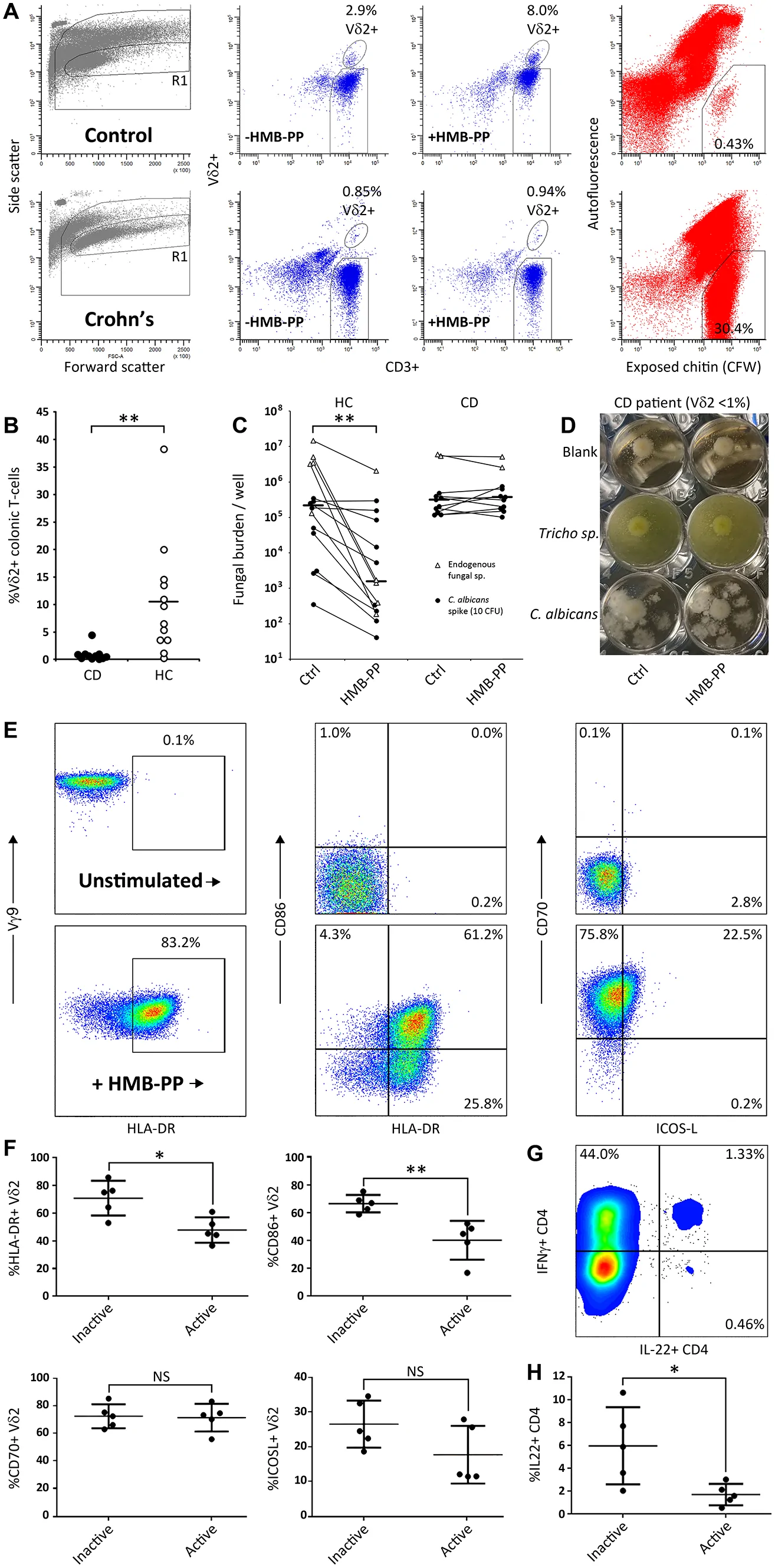
Vδ2+ T cell-mediated fungal suppression is impaired in patients with Crohn’s disease. Stimulation of healthy gut tissue with 10nM HMB-PP for 3 days at 37 °C triggered expansion of mucosal Vδ2+ cells (n = 13, mean 10.56% of T-cell pool), and restricted growth of endogenous fungi or spiked-in *C. albicans* reference strain SC5314, whereas biopsies from CD patients contained only trace Vδ2+ cells (n = 11, mean 0.85%) and failed to restrict fungal growth **(A-D)**. Representative FACS plots in **(A)**, grouped colonic Vδ2+ T-cell expansion data in **(B)**, quantification of fungal burden in **(C)**, and example cell culture photo in **(D)**. In a separate cohort of CD patients (n = 10), blood Vδ2+ T-cells exposed to HMB-PP for 4 days acquired a co-stimulatory profile (**E**; inactive CD example), but were significantly less responsive in active compared with inactive disease (n = 5 per group), as indicated by lower expression of antigen presenting markers HLA-DR and CD86 after stimulation **(F)**, as well as poor induction of IL-22 in allogeneic naïve CD4+ responder T-cells from healthy donors **(G, H)**. Bars indicate mean values (with standard error in **F** and **G)**. *p < 0.05, **p < 0.01 by *T* test **(B, F, G)** or Wilcoxon Signed Rank Test **(C)**. The data underlying this Figure can be found in https://doi.org/10.5281/zenodo.23064172.

### Diversity of mucosal *C. albicans* strains in healthy donors and patients with Crohn’s disease

We next explored the range of intestinal fungi and virulence profiles in gut tissue with intact or impaired Vδ2+ T-cell suppression, among which *C. albicans* was the species isolated most frequently (Fig 3A and 3B). Increased gut burden of *C. albicans* has been a consistent finding in previous studies of the IBD-associated microbiota [32], and a recent analysis of mucosal lavage strains linked hyphal formation with mouse macrophage damage in vitro and colonic injury in vivo [9]. We therefore assessed fungal cell wall composition, filamentation capacity, expression of CLYS-encoding gene *ECE1*, and epithelial cell damage inflicted by our collection of human gut biopsy-derived *C. albicans* strains.

**Fig 3:**
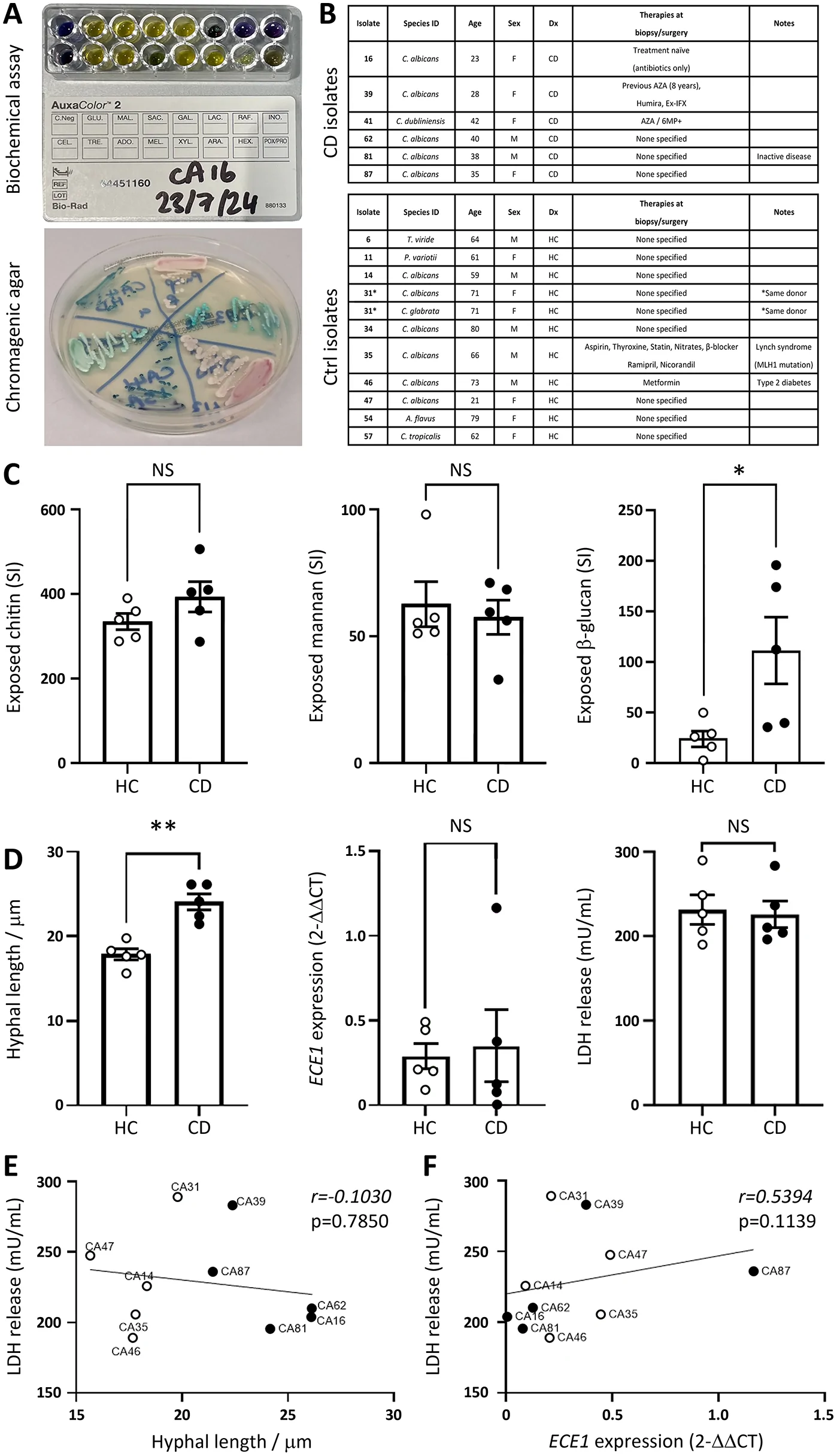
Biodiversity of *C. albicans* strains in healthy and inflamed human intestine. Fungi isolated from human colonic tissue were identified using a range of morphological and biochemical criteria, including AuxaColor2 and CHROMagar *Candida* assays **(A)**. *C. albicans* was the most prevalent species in both healthy control and CD tissue cultures **(B)**. *C. albicans* gut strains were next assessed to determine cell wall composition, extent of filamentation on plastic, *ECE1* gene expression, and LDH release as a measure of Caco-2 epithelial cell damage. Staining of immunogenic ligands in the yeast cell wall revealed increased levels of β-glucan exposure among *C. albicans* strains from CD patients (*p = 0.0317 by Mann-Whitney test), while chitin and mannan exposure were comparable between groups **(C)**. CD-derived strains displayed increased hyphal length after 2 h culture (**p = 0.0079 by Mann-Whitney test), whereas qPCR analysis of *ECE1* mRNA indicated comparable expression levels between cohorts **(D)**. Fungal co-culture with Caco-2 cells confirmed similar damage induction by *C. albicans* strains from HC vs. CD donors under basic conditions in vitro. Further assessment using linear regression did not directly correlate epithelial damage with either germ-tube/hyphal length **(E)** or *ECE1* mRNA expression level **(F)**. Parts C-F show *C. albicans* strains from donors <75 years-old to limit the impact of advanced age (which in primates impacts known influences on fungal biology including gut pH, motility, oxygen level, and IgA secretion) [82]. HC-derived *C. albicans* strains are shown as white circles (n = 5), and strains from CD patients as black circles (n = 5). Bars in C and D indicate mean and standard error. The data underlying this Figure can be found in https://doi.org/10.5281/zenodo.23064172.

Staining of immunogenic ligands in the yeast cell wall revealed increased β-glucan exposure among *C. albicans* isolates from CD patients, but did not detect any significant differences in levels of exposed chitin or mannan (Fig 3C). We next designed universal PCR primers (based on 350 *C. albicans* genome sequences) to detect regions of the *ECE1* gene that are conserved across strains. All intestinal *C. albicans* isolated from mucosal tissue cultures expressed *ECE1* mRNA, which was detected at highly variable levels in vitro that did not reflect patient donor status (Fig 3D). Importantly, *ECE1* transcription is just one step in the complex process of CLYS production, processing, and delivery via the hyphal tip [33,34], so we next quantified hyphal extension and epithelial damage in direct co-culture assays. These analyses revealed that CD-derived strains exhibited greater hyphal extension in vitro, although this was not significantly correlated with gut epithelial cell damage (Fig 3E). Similarly, *ECE1* mRNA expression levels alone were unable to predict epithelial cell injury in vitro (Fig 3F), as also reported in other studies of clinical isolates [35]. Together, these data reveal that human intestinal *C. albicans* strains display diverse patterns of filamentation, hypha maintenance, and *ECE1* expression in vitro. These differences likely give an incomplete profile of virulence in vivo, where gut microenvironmental factors exert a major influence on fungal behaviour.

### Gut-adapted *C. albicans* phenotypes are shaped by genetic and microenvironmental factors

To better understand drivers of *C. albicans* phenotypic diversity in human gut, and enhanced generation of invasive hyphae by CD isolates, we next employed whole genome sequencing of the mucosal tissue-derived strains. We observed a wide range of phylogenetic relationships that spanned multiple different clades (S2 Fig), consistent with reports that diverse genetic profiles are compatible with mammalian gut colonization [36]. We then focused our analysis on 8 genes that control the core filamentation response (*ALS3, DCK1, ECE1, HGT2, HWP1, IHD1, RBT1,* and *orf19.2457*) [37], which revealed that SNPs in one or more of these regulators were present across all gut tissue-derived strains (Fig 4), with no significant difference in frequency between isolates from healthy donors or CD patients (Fisher's exact test, p = NS). These data suggest that DNA sequence variants alone are unlikely to account for enhanced filamentation of *C. albicans* strains in CD, suggesting that epigenetic adaptation to the gut microenvironment plays a major role in shaping phenotypes [38,39].

**Fig 4:**
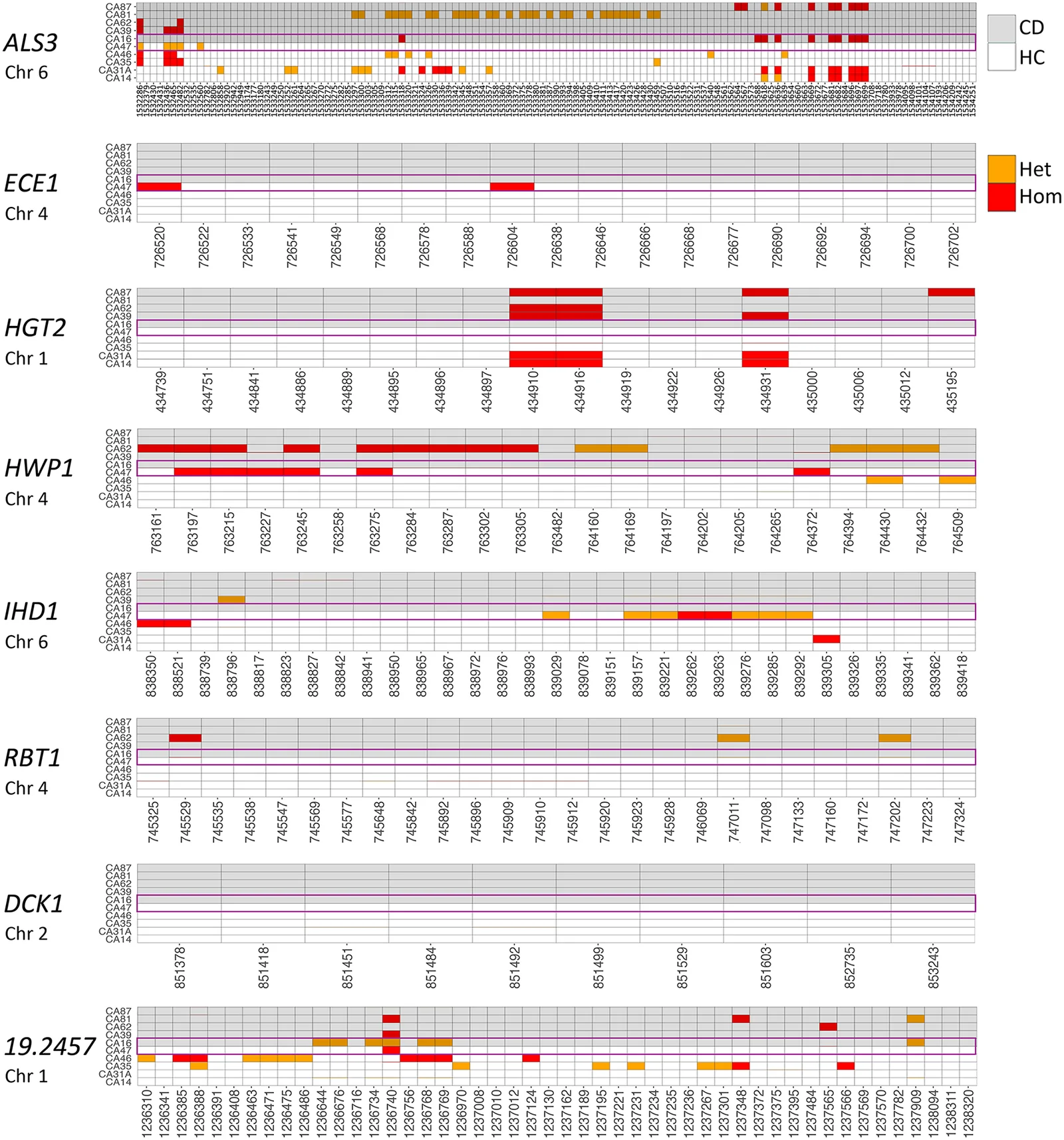
Human gut-adapted *C. albicans* strains exhibit core filamentation gene mutations. Gut-adapted strains were subjected to whole genome sequencing by Illumina methodology and variants were called against the *C. albicans* reference genome (Ca22) using the GATK *HaplotypeCaller* pipeline. All gut tissue-derived strains featured gene mutations in one or more regulators of the core filamentation response [37] (Orange = heterozygous, Red = homozygous). Purple boxes highlight SNPs in exemplar strains HC-CA47 and CD-CA16 selected for subsequent functional assays. X-axis shows SNP position on corresponding chromosome.

Among many gut physiological factors that can influence fungal growth in vivo, oxygen restriction is a key mechanism of *C. albicans* control and also closely linked with mucosal IL-22 responses [40,41]. Given that *C. albicans* phenotype was highly consistent between isolates within each patient group, we next selected one exemplar strain per cohort and used pSILAC proteomics to perform deep characterization of yeast-like and hyphal forms under ‘gut-like’ hypoxic conditions. For this analysis, we selected strain CA16 from a 23 year-old female patient with severe CD who had received only antibiotic therapy, and strain CA47 from a 21 year-old healthy female on no medication (Fig 5A and 5B). In addition to being closely matched for donor age, sex, and minimal treatment, CA16 and CA47 displayed typical features of their respective cohorts and diametrically opposite filamentation patterns in vitro (Fig 3E). Among a list of >2500 fungal proteins identified (S2 Table), this analysis revealed starkly different profiles for each isolate (S3 Fig), with CD-derived strain CA16 being significantly more responsive to environmental change (>750 proteins upregulated in hypoxic culture versus <100 for HC strain CA47; p < 0.05 by one-way ANOVA). These data align with reports that commensal organisms are less sensitive than pathogens to varying levels of heat and oxygen, which are typically stable in the natural host environment [42]. Indeed, key processes in hypoxic CA16 included ‘filamentous growth’, ‘negative regulation of immune response’, and ‘sequestering of zinc ion’, which are key determinants of *C. albicans* translocation across intestinal barriers [43] (S4 Fig). Strikingly, filamentation was not a highlighted pathway in hypoxic CA47, which was instead characterized by ‘symbiont modulation of host immunity’.

**Fig 5:**
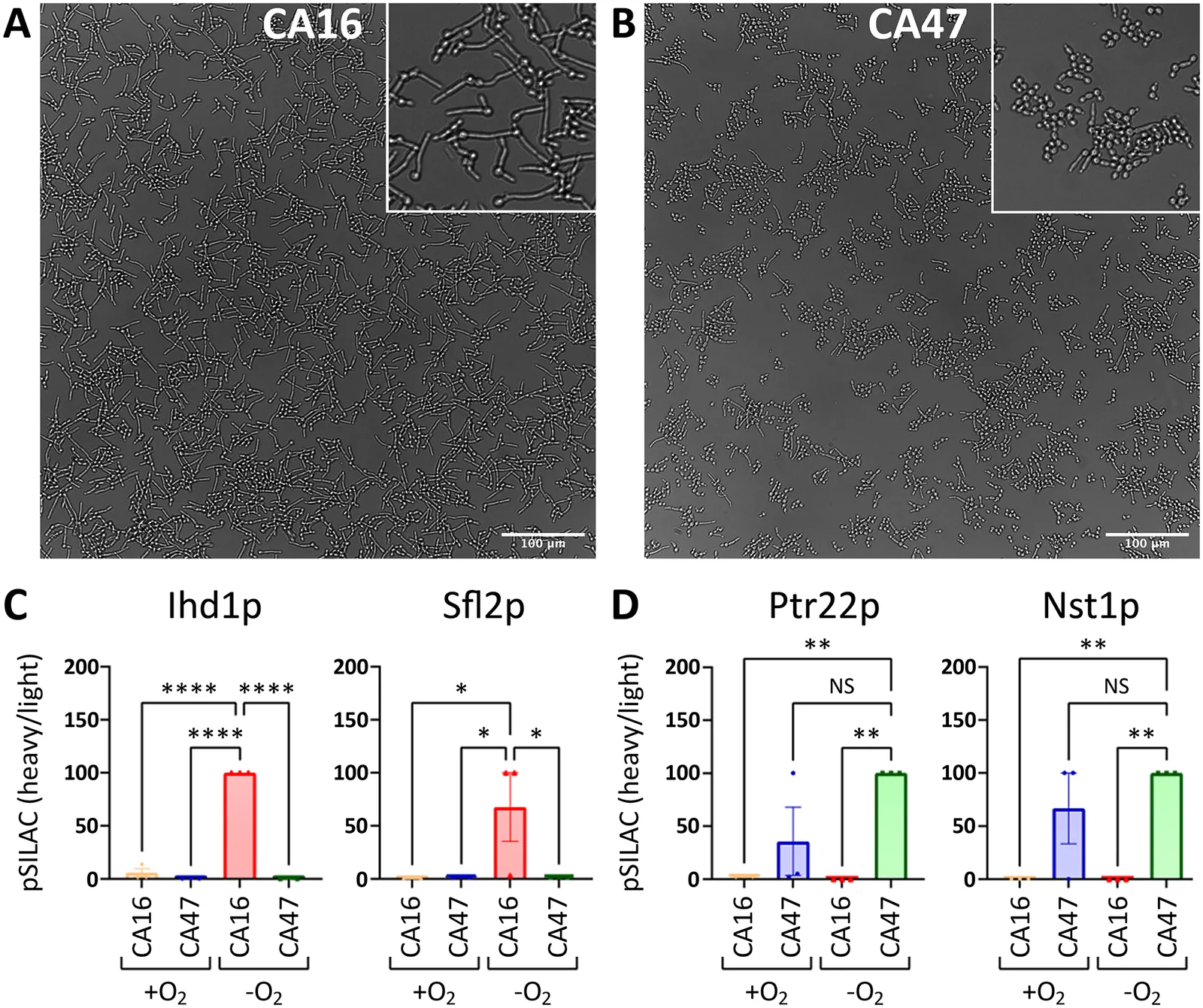
Hypoxia modifies the biology of gut-adapted yeast and filamentous *C. albicans.* Representative CD-derived strain CA16 **(A)** and HC-derived strain CA47 **(B)** were cultured in YNB medium (supplemented with sucrose and ammonium sulphate) in either normoxia or hypoxia (~98% N_2_, ~ 2% O_2_) overnight, then changed to pSILAC medium (R/arginine and K/lysine-free RPMI medium supplemented with heavy R6 and K8 amino acids) for a further 3 h prior to analysis by pSILAC proteomics. In low-oxygen cultures resembling human gut conditions, the top 10 differentially expressed proteins included hyphal regulators Ihd1p and Sfl2p, which were actively translated only by CD-derived strain CA16 **(C)**. HC yeast CA47 instead expressed mediators of niche-specific stress tolerance, including Ptr22p and Nst1p **(D)**. Scale bars indicate 100 μM **(A and B)**. Bars in C and D indicate mean and standard error of n = 3 biological repeats (*p < 0.05, **p < 0.01, ****p < 0.0001 by ANOVA). The data underlying this Figure can be found in S2 Table.

Together, these data confirm that the intestinal environment exerts a major influence on *C. albicans* phenotypes, with CD-derived strain CA16 seemingly poised for filamentation under ‘gut-like’ hypoxic conditions. Among the proteins most strongly expressed by CA16 during low-oxygen culture was core filamentation regulator Ihd1p (Fig 5C), whereas this hyphal-specific protein was barely detected in CA47, which featured numerous SNPs in the corresponding gene (Fig 4). CA16 also upregulated the hyphal transcription factor Sfl2p, which senses the local environment to promote mouse gastrointestinal infection [44,45], but despite an identical gene sequence CA47 failed to express this virulence-linked protein. Instead, CA47 was enriched in regulators of nutrient uptake and tolerance of tissue-specific variables including iron, oxygen, heat, and osmotic stress (Fig 5D), as well as epigenetic adaptation to the mammalian gut, e.g., ‘white-opaque’ switch Med9p [39,46] (S2 Table). Together, these data confirm that niche-specific factors exert a strong influence on the relative pathogenicity of genetically diverse *C. albicans* strains in human intestine, with likely major consequences for mucosal immunity and inflammation.

### Host phagocyte responses to intestinal *C. albicans* are modified in Crohn’s disease

We next explored the potential host immune impact of *C. albicans* escape from suppression by intestinal Vδ2+ T-cells, as well as the consequences of increased filamentation among CD isolates. For this, we focused on neutrophils and monocyte-macrophages, which are critical mediators of antifungal immunity and highly enriched in the mucosa of colitis and CD patients respectively. Key functions of these lineages include neutrophil engulfment of fungi and hyphal destruction via NETosis [47], alongside macrophage phagocytosis and folding of developing hyphae [48]. We therefore assessed the induction of NETosis by our human gut-derived *C. albicans* strains, as well as monocyte-derived macrophage (MoMΦ) susceptibility to CLYS exposure, strain-dependent damage, and IL-1β induction in vitro.

When co-incubated with healthy donor blood neutrophils, CD-derived CA16 triggered significantly higher levels of NETosis relative to HC-derived CA47 (Fig 6A), confirming that neutrophils can discriminate between yeast and hyphal morphology of human gut-adapted strains [47]. Intriguingly, divergent NETosis responses to CA16 and CA47 were lost in assays using blood neutrophils from CD patients (Fig 6B), reflecting significant functional impairment of this compartment in disease (S5 Fig) [49]. When we performed similar assays using healthy blood MoMΦ, these cells released only limited amounts of IL-1β upon challenge with HC strain CA47, which was indistinguishable from exposure to a CLYS toxin-only control (which lacks potency in the absence of hyphal filaments) (Fig 6C). In contrast, CD-derived strain CA16 was a strong inducer of MoMΦ-derived IL-1β, which was released at comparable levels to macrophages challenged with hyper-virulent reference strain SC5314. Similar patterns of IL-1β release were observed when using MoMΦ generated from patients with CD (Fig 6D), although the efficiency of fungal clearance was markedly reduced irrespective of the strain used during co-culture assays. Importantly, the more virulent profile of strain CA16 was unable to escape suppression by HMB-PP-activated Vδ2+ T-cells when spiked into healthy colonic biopsy cultures (S6 Fig). Together, these data indicate that key human leukocytes mediating antifungal immunity and mucosal inflammation display altered responses to *C. albicans* strains that persist in CD intestine in the absence of Vδ2+ T-cell control.

**Fig 6:**
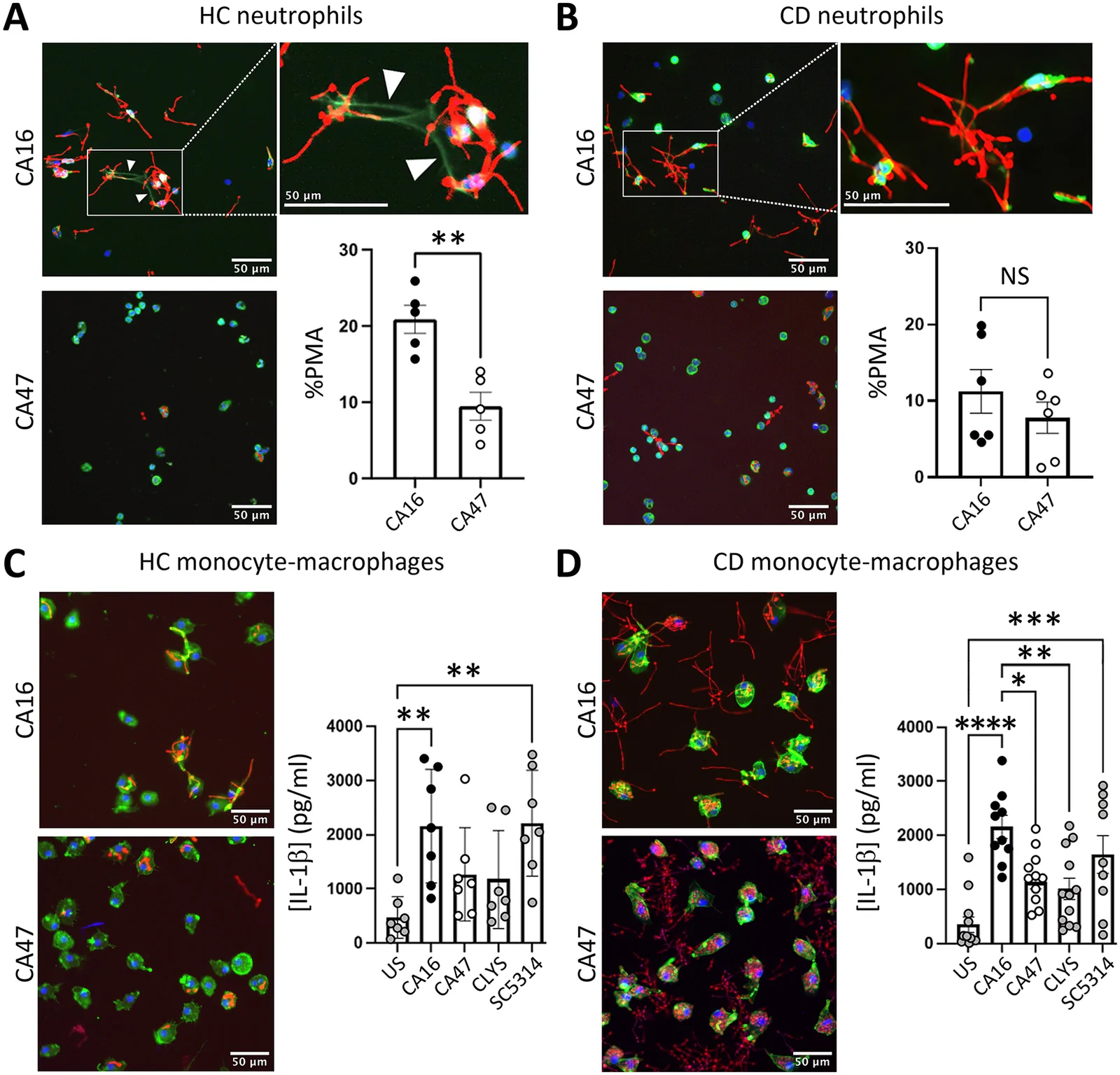
Phagocyte responses to human intestinal *C. albicans* strains are modified in CD. Peripheral blood neutrophils (CD66b+CD16+) were isolated and incubated with *C. albicans* strains (red) for 3h before staining with antibodies against MPO (green), and calprotectin (grey), together with Hoechst nuclear stain (blue). Production of NET-like structures (NLS) was quantified relative to a PMA positive control using SYTOX Green assays. **(A)** Neutrophils from HC donors (n = 5) produced more NLS (white arrows) when challenged with CA16 compared with CA47 (p = 0.0079; unpaired *t* test and Mann Whitney test). **(B)** Neutrophils from CD patients (n = 6) produced similar levels of NLS when exposed to either CA16 or CA47 (p = 0.4848; unpaired *t* test and Mann Whitney test). Data shown as mean±SEM. Similarly, blood CD14+ monocytes were differentiated into macrophages by 7-day culture with M-CSF and primed with 50 ng/mL LPS for 2h before challenge with strains CA16, CA47, or SC5314 (all MOI:5) or synthetic CLYS for 5h. IL-1β release was then assessed by ELISA. Representative images show *C. albicans* (red), Hoechst-stained nuclei (blue), and phalloidin-stained actin (green). When cultured with macrophages from healthy donors (n = 7), CD strain CA16 induced comparable levels of IL-1β release to hyper-virulent reference strain SC5314, whereas HC strain CA47 was indistinguishable from the CLYS-only control **(C)**. Similar patterns were obtained using macrophages from CD donors (n = 10), although fungal burden per well appeared higher **(D)**. Scale bars indicate 50 μM. Graph bars show mean and standard error. *p < 0.05, **p < 0.01, ***p < 0.001, ****p < 0.0001 by *T* test **(A and B)** or ANOVA **(C and D)**. The data underlying this Figure can be found in https://doi.org/10.5281/zenodo.23064172.

### Rapid fungal adhesion and invasion of the gut barrier in a human ‘organ-on-a-chip’ model

In typical mouse models of *C. albicans* colonization, strain-dependent kinetics of epithelial binding and invasion depth exert a critical influence on *C. albicans* commensalism versus tissue damage and expulsion from the gut [35]. While yeast morphotypes tend to outcompete hyphal cells in antibiotic-treated mouse gut [50,51], strains able to form hyphae appear significantly fitter when the bacterial microbiota is intact [52], further supporting a major influence of gut environmental variables on *C. albicans* phenotypes in vivo. Indeed, our proteomic data suggested variable tissue adhesion/invasion potential of strains CA16 and CA47 under ‘gut-like’ conditions in vitro, but it was unclear whether this would apply under dynamic conditions in the human intestine. Since *C. albicans* does not naturally colonize mouse intestine, we instead developed a human ‘gut-on-a-chip’ model to explore fungal adhesion/invasion dynamics in the relevant ‘in vivo’ setting. We first transformed exemplar clinical strains CA16 and CA47 with pENO1-iRFP-NATr fluorescent reporters (as described previously) [53] then assessed fungal binding and translocation across the model gut barrier when subject to physiologically relevant liquid flow and peristaltic stretch (Fig 7A–7E).

**Fig 7:**
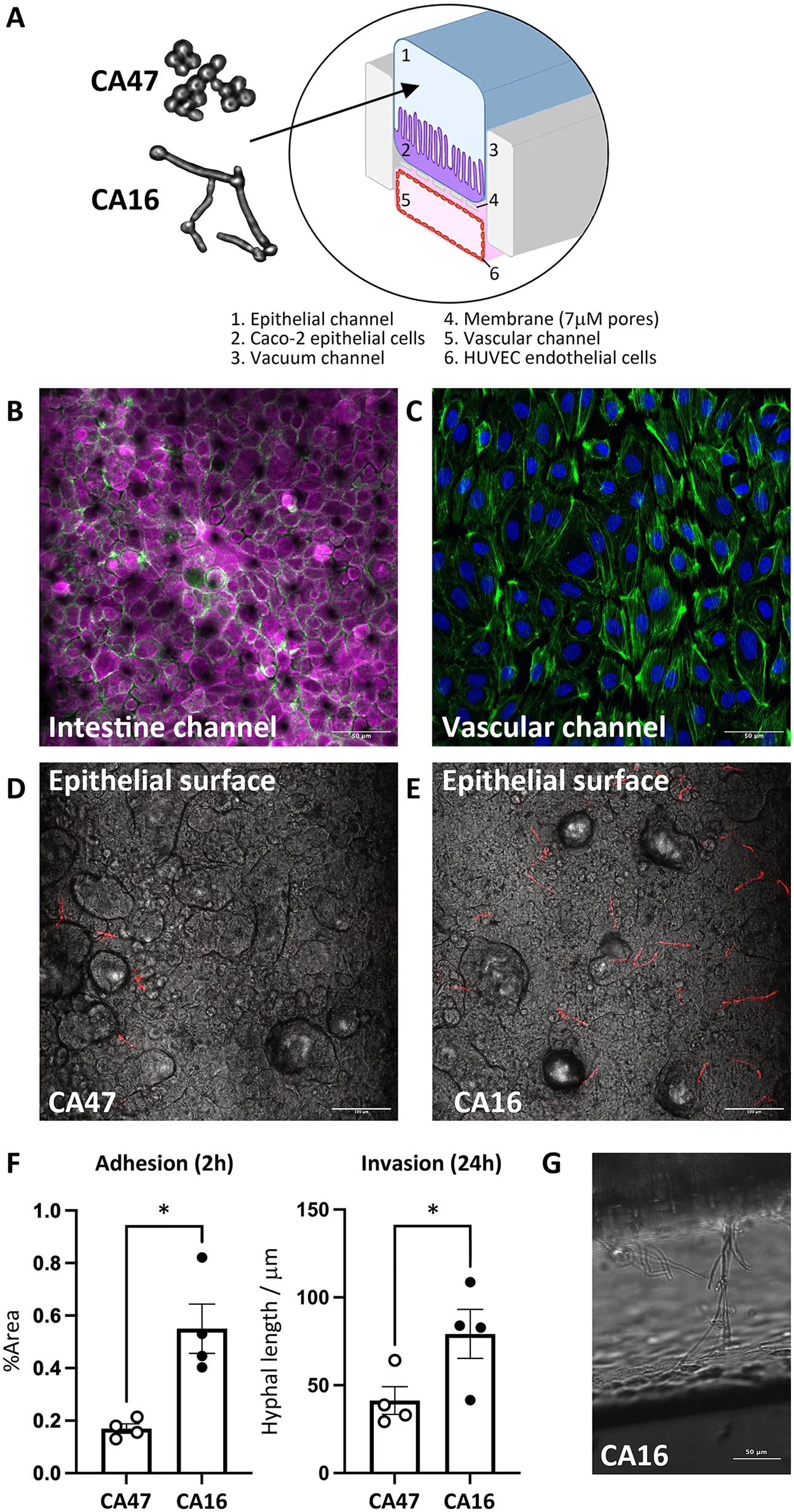
Rapid fungal adhesion and invasion of a dynamic human ‘gut-on-a-chip’ model. Schematic of the gut-on-a-chip model formed of two parallel channels separated by a porous membrane and subjected to physiologically-relevant fluid flow and stretch forces **(A)**. The intestinal channel contained Caco-2 epithelial cells which formed continuous tight junctions and 3D villous-like structures under peristaltic motion **(B)**. The underlying vascular channel was coated with HUVEC cells to create a model blood vessel **(C)**. Human gut-adapted *C. albicans* strains expressing pENO1-iRFP-NATr fluorescent reporters were then introduced to the model gut lumen for assessment of host tissue binding and invasion potential. Early-stage adhesion was measured as %area bound by fluorescent *C. albicans* in top-down images of the epithelial surface assessed after 2 h incubation (using Fiji software), revealing limited binding of HC yeast CA47 **(D)** relative to filamentous CD strain CA16 **(E)**, which was significantly more adhesive by unpaired *t* test (**F**; p = 0.0074, n = 4 per group). When hyphal protrusions into vascular channel Z-stack images were measured at 24 h post-infection, CD strain CA16 was significantly more invasive (p = 0.0412, n = 4 per group), and displayed extensive growth into the model blood vessel (**G**; magnification x20). Scale bars indicate 50 μM **(B, C, G)** or 100 μM **(D and E)**. Bars in F indicate mean and standard error (*p < 0.05). The data underlying this Figure can be found in https://doi.org/10.5281/zenodo.23064172.

Tissue 3D structure and peristaltic motion have been shown to modify invasion of the primate-specific bacterial pathogen *Shigella flexneri* into human gut models [54]*,* and key genes associated with fungal adhesion to biological surfaces can be expressed differentially between strains as early as 90 min after contact [55]. We therefore assessed epithelial binding 2 h after spiking human gut-adapted strains CA16 and CA47 into our intestine-on-a-chip system under conditions of physiological flow and mechanical stretch (S1 Video). When quantified by fluorescent imaging, commensal-like HC strain CA47 displayed only low-level adhesion to the gut surface after 2 h incubation, with limited filamentation in situ. In contrast, CD-derived strain CA16 exhibited rapid binding to the gut surface with extensive hyphal growth, followed by significant translocation into the underlying vascular channel within just 24 h (Fig 7F, 7G, and S2 Video). These functional assays were consistent with our genotypic, proteomic, and immunological data suggesting that CD-derived strain CA16 is more pathogenic than healthy control-derived strain CA47. Importantly, both strains were capable of binding to the gut epithelium and eventually invaded the model vasculature even when subjected to dynamic physiological forces. Future work will therefore require the development of more advanced organ chip models, replete with multiple lineages of human leukocytes, which can be used to determine how these fungal dynamics are modified by host immunity in health and inflammatory settings ‘in vivo’.

## Discussion

Here we report that bacterial metabolite HMB-PP can activate human intestinal Vδ2+ T-cells to suppress fungal pathobionts in healthy gut tissue via an IL-22-dependent mechanism that is dysregulated in Crohn’s disease (CD). These data are in-line with previous reports that commensal metabolites can modify host immunity to influence competition between microbial strains, species, and even kingdoms [3–6], whereas defects in BTN/L regulation of the human γδ T-cell compartment have been linked with severe CD phenotypes [56].

Intestinal γδ T-cells play a critical role in gut barrier defense [57], where IL-22 appears crucial in the early response to adherent-invasive pathogens [23–58]. While T-cells producing IL-17 are highly enriched during human gut inflammation, those producing IFNγ and IL-22 are significantly depleted, in correlation with relative loss of the bacterial phylum Firmicutes [31] (which includes numerous HMB-PP-producing genera) [18]. Anaerobic Firmicutes have also been shown to promote *C. albicans* expulsion from the mouse gut via activation of HIF-1α, which cross-talks with AhR to promote T-cell expression of IL-22 but not IL-17 [59–61]. Similarly, we previously identified that bacterial activation of Vδ2+ T-cells in human gut tissue selectively induces *AHR* and IL-22 [20], which in the current study emerges as a potent mechanism of fungal suppression in healthy gut but not CD intestine. Indeed, blood and biopsies from CD patients were characterised by poor Vδ2+ T-cell sensitivity to HMB-PP and trace cell numbers in mucosa, accompanied by rapid outgrowth of endogenous fungal species [29]. These findings align with reports that azathioprine therapy selectively depletes Vδ2+ T-cells in CD [29], and AhR/HIF-1α/IL-22 signalling is impaired in gut biopsies from IBD patients [62], whereas provision of AhR ligands or drugs that restore epithelial hypoxia can limit *C. albicans* growth [41]. It is possible then that failing hypoxic control and loss of anaerobic bacteria in the CD intestine disrupts Vδ2-mediated fungal suppression, allowing more virulent *C. albicans* strains to persist. At present, we cannot resolve whether loss of fungal control in CD is due mainly to functional impairment or numerical deficits in the Vδ2 T cell compartment, although our data clearly demonstrate that both features are present.

*C. albicans* isolates from CD gut tissue were significantly more filamentous, indicating greater potential for host tissue invasion, but *ECE1* gene expression and epithelial damage profiles could not be distinguished from control strains under basic culture conditions in vitro. Mutations in core filamentation genes were frequent but diverse among strains, which displayed both yeast and hyphal morphologies that broadly aligned with donor inflammatory status rather than genotype. Consistent with this, *C. albicans* colonization of mouse cecum is accompanied by high expression of hyphal-linked gene *ECE1*, despite yeast cell morphology being the dominant growth form in this setting [63]. These findings suggest that *C. albicans* adaptation to the mammalian intestine is a complex process shaped not only by genotype, but also epigenetic effects of the gut microenvironment and host immune variables.

Human inborn errors of Th17 immunity are strongly linked with mucocutaneous candidiasis, but affected patients are typically also deficient in IL-22 and IFNγ, with pathology varying significantly between body sites [64,65]. These data suggest that the optimal balance of Th1/Th17 immunity needed to prevent fungal infection varies by anatomical location, epithelial structure, and local environmental factors [12,13]. In human gut, *C. albicans*-responsive cells are strikingly enriched within the Th17 pool and expand during mucosal inflammation, which in CD patients is characterised by the further generation of Th1 cells that cross-react with other fungal species [66,67]. Variable host engagement of Th1 and Th17 responses may in-turn alter *C. albicans* levels and phenotypes in the human intestine, with gut burden known to differ significantly even between healthy adults [8]. Local conditions are also likely to play a role, since the healthy gut epithelium is maintained in a state of physiological hypoxia, which our proteomic analysis revealed to induce starkly different profiles for CD strain CA16 compared with HC strain CA47. Key points of divergence included regulators of filamentation such as IHD1, and responsiveness to tissue-specific variables including iron, oxygen, heat, and osmotic stress. Consistent with these data, mice fed a conventional diet are generally refractory to gut colonisation with virulent reference strains such as SC5314 that readily form hyphae, but persistence can be impacted by sugar intake, antibiotic treatment, and mucosal inflammation [52,68]. Indeed, antibiotic treatment also increases risk of new onset CD in humans [69], where hyphal strains of *C. albicans* appear more prevalent in mucosal lavage [70], but the downstream impact on host immunity and inflammation have remained unclear.

Many IBD risk genes are now recognised to play important roles in host-fungal interactions [71]. In particular, *CARD9* protects mice against colitis by promoting expression of IL-22 [72], which can be triggered by *C. albicans* proximity to the intestinal epithelium [22]. Similarly, *NOD2* expression is largely restricted to the monocyte-macrophage lineage [73], which plays a crucial role in fungal sensing at the gut barrier [70,74–76]. We observed greater levels of neutrophil NETosis and macrophage IL-1β release in response to *C. albicans* derived from CD gut tissue, suggesting increased virulence potential in disease. A key mechanism of host defence against virulent *C. albicans* is thought to require *CARD9*^+^ macrophages that induce antifungal IgG [76] and secretory IgA which preferentially targets hyphal cells [70]. In healthy gut this disadvantages pathogenic strains and favours commensal yeast that have mutated hyphal genes [46,50], whereas sIgA selectivity for hyphal targets is impaired in CD [70], perhaps allowing for persistence of more invasive strains. We therefore developed a custom ‘gut-on-a-chip’ model to assess the extent to which *C. albicans* isolates from HC and CD gut can bind and damage human host tissues under relevant physiological conditions of peristaltic fluid flow and mechanical stretch ‘in vivo’. Consistent with our phenotypic, genomic, and proteomic data, *C. albicans* isolated from CD tissue displayed significantly greater ability to adhere and invade the model tissues.

It is increasingly clear that a complex range of variables can impact host-fungal interactions in the human intestine, including quantity and diversity of bacterial competition, range of nutrient sources available, and local oxygen gradients. Considering major differences between mouse and primate gut physiology, resident microbiota, and immune profiles, it is perhaps unsurprising that *C. albicans* can be a universal colonizer of humans but not rodents. By extension, natural human hosts may engage mechanisms of fungal control that are not present in rodent systems, such as our current finding that mucosal Vδ2+ T cells can suppress growth of intestinal *C. albicans*. Given that both the target microbe and host immune mediator of this axis are absent in mice, alternative model systems will be needed to fully understand this biology and perhaps uncover further new aspects of human tissue defence against fungal infections.

## Materials and methods

### Ethics statement

Collection of clinical data and biological specimens was performed under the NIHR Research Tissue Bank approval ‘BIORESOURCE STUDIES OF THE DIGESTIVE SYSTEM IN CHILDREN AND ADULTS’ (REC Number: 15/LO/2127). The study was conducted according to the principles expressed in the Declaration of Helsinki. All volunteers gave written informed consent prior to inclusion.

### Study participants

Peripheral blood and colonic biopsies were obtained from patients undergoing endoscopy as part of routine clinical care to assess CD activity, or for colorectal cancer screening or investigation of rectal bleeding but with no significant endoscopic abnormalities (controls). Larger mucosal tissue samples were obtained from patients undergoing surgical resection for colorectal cancer, non-inflammatory intestinal motility disorders, or severe CD (whereby a consultant pathologist dissected a window of mucosal tissue for research, which was then divided into biopsy-sized pieces prior to use in the lab). Both cohorts included equal numbers of male and female donors (1:1 ratio), while the control group (n = 28, mean age 46; range 21–80) was significantly older than the CD group (n = 43, mean age 31; range 16–60) reflecting typical demographics of the gastroenterology clinic (p = 0.0005 by Welch’s *t* test). All samples were collected from ascending colon unless indicated otherwise: either macroscopically healthy tissue in control donors, or actively inflamed sites in patients with CD (see S1 Table for full cohort demographics, clinical details/specimen types, and fungal isolates as relevant).

### Peripheral blood cells

Human whole blood was collected into sodium-heparin vacutainers (BD Bioscience) and used for PBMC isolation (below) or directly labelled with monoclonal antibodies for 15 min at room temperature prior to lysing red blood cells by addition of Optilyse C (Beckman Coulter, Bucks). Labelled cell suspensions were washed twice in cold FACS buffer (PBS containing 2% fetal calf serum, 0.02% sodium azide, 1 mM EDTA) and fixed in 1% paraformaldehyde prior to analysis by flow-cytometry.

### PBMC isolation

Peripheral blood was diluted 2:1 with room-temperature RPMI and the PBMC fraction was isolated by density gradient centrifugation over Ficoll-Plaque PLUS (GE Healthcare). Mononuclear cells were aspirated from the interface and viability was determined by Trypan Blue dye exclusion (Gibco).

### Neutrophil isolation

Blood was collected into K-EDTA tubes and processed using EasySep Direct Human Neutrophil Isolation Kits (StemCell Technologies, Inc.) according to the manufacturer’s protocol. A total of 75 x 10^3^ neutrophils in 50 µL phenol-red free RPMI-1640 medium (supplemented with 1% HEPES and 1% FBS) were added into each chamber of a 96-well flat-bottom plate and allowed to settle for 30 min at 37 °C, 5% CO_2_. Next, 50 µL of pre-diluted stimuli were added to replicate wells of neutrophils, followed by incubation for 3 h at 37 ^°^C, 5% CO_2_ (stimuli included 10^6^/mL *Candida*, 200 nM PMA, 5–75 µM CLYS toxin, 5 µM Ionomycin, 1 µg/mL LPS). After incubation, supernatants were removed and cells were either stained with SYTOX Green or fixed immediately by addition of 4% PFA overnight. The following day, supernatant was discarded, cells were washed 3 times with PBS, and a final volume of 200 µL PBS was added for sample storage until analysis.

### Monocyte-derived macrophages

Positive selection of CD14+ PBMCs was carried out using the EasySep Human CD14 Positive Selection Kit II (StemCell Technologies, Inc.) according to the manufacturer’s protocol. Primary blood monocytes were adjusted to a final concentration of 3 x 10^5^ per mL in complete medium (Dutch-modified RPMI-1640 supplemented with 10% FBS, 1% Penicillin-Streptomycin, 1% L-glutamine, 50 ng/mL M-CSF) and cultured in 96-well plates at 37 °C, 5% CO_2_ for 7 days (half the culture volume was replaced on day 3 with fresh medium containing 50 ng/mL M-CSF). Macrophages were harvested by adding medium containing 1 mM EDTA and placing on ice for 10 min.

### Colonic organ culture

The mucosal tissue culture method was modified from Mahida et al. [77] and has been described in detail previously [29,78,79]. In brief, colonic biopsies or equivalent sized pieces of surgically-resected mucosa were washed in 1 mmol/L dithiothreitol (Sigma-Aldrich) and cultured in flat-bottom 24 well plates in complete medium (Dutch-modified RPMI-1640 medium, 10% FCS, 2 mM L-glutamine, 100 u/mL penicillin, 100 μg/mL streptomycin, 25 µg/mL gentamicin, recombinant human IL-2 [30 u/mL] and IL-15 [20 ng/ml]) in the presence or absence of 10nM bacterial phosphoantigen HMB-PP (Echelon Biosciences) and blocking antibodies (ultra-LEAF purified anti-IFN-γ [clone B27] from BioLegend, or anti-IL22 [IL22JOP] from ThermoFisher) for up to 3 days at 37°C, 5% CO_2_. After 3 days, intact tissues were discarded and egressed leukocytes were labelled with mAb and/or fungal content was quantified by staining with calcofluor white (CFW; 25 μg/mL for 15 min) prior to analysis by flow cytometry. Alternatively, the cultures were resuspended into 96-well round-bottom plates in fresh medium for further incubation/functional assays.

### Neutrophil SYTOX green assay

SYTOX Green was used to quantify cells undergoing NET formation. After 3 h incubation with the indicated stimuli, SYTOX Green was added at a concentration of 1 µM and fluorescence was measured immediately using a CLARIOStar plate reader at excitation 500 nm, emission 528 nm. Each stimulus was repeated in triplicate and average values were recorded as mean fluorescence. Total %NET release was calculated by subtracting background fluorescence signal (medium containing SYTOX Green only) from the values obtained, and then expressed relative to a PMA positive control (determined as 100% NET release).

### Neutrophil immunofluorescent staining

Neutrophils were permeabilized with a solution of 0.1% sodium citrate and 0.1% Triton X-100 in PBS for 10 min at 4 °C. After washing 3x with PBS, cells were blocked with 3% BSA and 10% serum (either donkey or goat depending on secondary antibodies used) and incubated for 60 min at 37 °C. After washing 3x with PBS, diluted primary antibodies (1:1000 H3Cit, 1:100 NE, 1:250 MPO, 1:200 Calprotectin) were added and cells incubated overnight at 4 °C. Cells were washed 3x with PBS before secondary antibodies were added (1:500 dilution) and incubated at room temperature in the dark for 2 h. Cells were washed 3x with PBS. Finally, Hoechst (1:10,000 dilution) was added and incubated for 10 min at room temperature. Cells were washed 3 times and resuspended in PBS for analysis.

### Macrophage infection assay

Macrophages were primed for 2 h with 50 ng/mL LPS in serum-free medium before challenge with the indicated *C. albicans* strains (MOI 5) or CLYS toxin prepared in sterile water (75 µM final concentration). After 5 h incubation the exhausted culture medium was collected and cytokine concentration determined using Human IL-1β ELISA MAX kits (Biolegend) according to the manufacturer’s instructions.

### Macrophage immunofluorescent staining

Macrophages were differentiated as described above before adding fluorescent reporter strains of *C. albicans* at MOI 1. After 3 h, plates were fixed with 4% PFA for 15 min at room temperature. Macrophages were permeabilized with a solution of 0.1% sodium citrate and 0.1% Triton X-100 in PBS for 10 min at 4 °C. After washing 3x with PBS, diluted AF488-Phallodin (1:500) was added and the cells incubated for 2 h at room temperature. Cells were then washed 3x with PBS before adding Hoechst (1:10,000 dilution) and incubating for 10 min at room temperature. Cells were washed 3 times and resuspended in PBS for analysis.

### γδ T-APC assays

Vγ9^+^ and Vδ2^+^ T cells (each >99% purity) were isolated from PBMC using anti-Vγ9-PECy5 (Beckman Coulter) or anti-Vδ2-PE mAb (BD Biosciences), combined with anti-PE microbeads (Miltenyi). γδ T-APCs were generated by co-culture with irradiated monocytes (50 Gy) at a 10:1 ratio, in the presence of 10 nM HMB-PP with or without 20 ng/ml IL-15. γδ T-APCs were cultured for 3–7 d and purified either by positive selection or cell sorting (purity >99%). Bulk CD4^+^ T cells (>95% purity) were isolated from PBMC via negative selection using the CD4^+^ T cell Isolation Kit (Miltenyi) then labeled with anti-CD4, anti-CD45RA, and anti-CCR7 mAb prior to sorting on a FACSAria II (BD Biosciences) to purify the naïve population (>99% CD4^+^ CD45RA^+^ CCR7^+^). γδ T-APCs were then irradiated at 12 Gy prior to co-culture with allogeneic CD4+ responder T-cells (1:10 ratio) for up to 7 d prior to analysis of phenotype and cytokine expression by flow cytometry (S1 Fig). For phenotypic analyses, cells were acquired on an eight-color FACSCanto II (BD Biosciences) and analyzed with FlowJo 10.1 (TreeStar). Anti-mouse Ab reactive beads were used to set compensation (Life Technologies). Single viable leukocytes were gated based on light scatter characteristics and exclusion of fixable Aqua Dead Cell Stain (Invitrogen), followed by gating based on fluorescence minus one controls.

### Cytokine staining

Cells were re-activated with PMA (10 ng/mL) and ionomycin (2 μM) in the presence of monensin (3 μM) for 4 h at 37°C, 5% CO_2_ prior to surface labelling. Re-activated cells were then permeabilized using Leucoperm reagents (AbD Serotec, Oxford, UK), labelled with anti-cytokine mAb, and fixed in 4% paraformaldehyde for analysis by flow-cytometry.

### Flow-cytometry

Monoclonal antibodies were; FITC-conjugated TCR Vδ2 (clone B6) and CD14 (HCD14), PE-conjugated ICOSL (2D3), PerCP-Cy5.5-conjugated CD3 (clone HIT3a), PE/Cy7-conjugated CD45RA (HI100), CD69 (FN50), CCR7 (G043H7), IFNγ (4S.B3), and CD16 (B73.1), APC-conjugated CD86 (IT2.2), AlexaFluor647-CD66b (G10F5), and Pacific Blue-HLA-DR (L243) from BioLegend UK. FITC-conjugated anti-CD70 (113-16), and PE/Cy7-conjugated anti-IL-22 (22URTI) were from eBioscience. APC-H7-conjugated CD4 (RPA-T4) HLA-DR (L243) were from BD Biosciences. PECy5-conjugated TCR Vγ9 (Immu360) was from Beckman Coulter. Labelled cells were acquired on a FACSCanto II flow-cytometer using FACSDiva 6.1.2 software (Becton Dickinson), and data were analyzed using WinList 9.0 (Verity Software House, Maine). Fungi were gated as CFW+ events relative to staining controls; PBMC only (negative), *C. albicans* reference strain SC5314 (positive), PBMC spiked with SC5314 (infected). Absolute numbers of CFW+ events were quantified using Flow-Count Fluorospheres (Beckman Coulter) with the formula;


$$\begin{array}{c} N_{absolute}=(N_{cellevents})/(N_{beadevents})*(C_{beads}*V_{\text{beads}})*DF \end{array}$$

where:

- $N_{\text{cell events}}$ = number of gated cell events
- $N_{\text{bead events}}$ = number of bead events
- $C_{\text{beads}}$ = certified bead concentration (beads/µL)
- $V_{\text{beads}}$ = volume of bead suspension added (µL)
- DF = dilution factor

### Fungal species identification

Fungal species were isolated during in vitro culture of mucosal tissue samples from HC donors and patients with CD. Initial fungal cultures were streaked onto BD ChromAgar *Candida* plates and identified using AuxaColor20 biochemical assays. Some species/strains underwent further confirmative identification by MALDI-TOF due to ambiguous results from standard profiling (performed by the Royal London Pathology Laboratory).

### Candidalysin (CLYS)

CLYS toxin (SIIGIIMGILGNIPQVIQIIMSIVKAFKGNK) was synthesised commercially (Peptide Synthetics, UK), solubilised in culture-grade water, and stored at 10 mg/mL at −20 °C until use.

### Candida filamentation assay

*Candida* isolates were cultured in YPD for 16–18 h at 30 °C then washed 3 x with PBS, resuspended in complete medium, and transferred to a 37 °C incubator. At the indicated time points, hyphal growth was stopped by fixation in 4% PFA for 15 min. Filament length was then determined using widefield microscopy (IN Cell Analyzer 2200, GE Healthcare) and analysis by ImageJ2 v2.3.0.

### Fungal cell wall analysis

*C. albicans* strains were inoculated into fresh YPD broth, incubated 16–18 hours at 30 °C to achieve stationary phase growth, and then washed into PBS. For mannan quantification, yeast were immediately stained with FITC-Concanavalin A (ConA; 100 µg/mL) for 30 min at room temperature in the dark. The labelled yeast were then washed with PBS and fixed with 4% PFA for 15 min at room temperature, after which fixative was removed by further washing in PBS. For β-glucan determination, the ConA-labelled cells were next stained with aniline blue (25 mg/mL) for 15 min at room temperature in the dark. For chitin determination, unstained yeast were first fixed with 4% PFA for 15 min at room temperature prior to addition of CFW (25 µg/mL) for 15 min. All samples were washed in PBS and re-suspended in 300 µL FACS buﬀer before acquisition on a BD FACSCanto II with FACSDiva 6.1.2 software (BD Biosciences) followed by analysis using WinList 9.0 (Verity Software House, Maine). Staining intensity for each cell wall component was calculated relative to unstained control yeast (expressed as staining index; SI).

### Quantification of ECE1 gene expression

Epithelial cells were infected with *Candida* species/strains for 24 h (MOI 0.01). Exhausted culture medium was collected, and non-adherent fungi were pelleted by centrifugation. Adherent fungi were rinsed with ice-cold PBS, loosened with a cell scraper, then added to the pelleted sample. Fungi were washed with 1 mL ice-cold PBS, resuspended in a final volume of 500 µL, and added to a cryo-vial containing approximately 1/3 volume of acid-washed glass beads (Thistle Scientific, 0.5 mm) for 4 rounds of bead beating at 4.5 m/s^2^ for 30 s using a FastPrep-24 system (MP Bio). Lysates were removed and RNA was extracted using a MasterPure Yeast RNA Purification Kit (Lucigen) according to the manufacturer’s instructions. Removal of contaminating gDNA was performed according to kit instructions. For t = 0 h control samples, RNA was extracted from 500 µL of each YPD fungal culture in the absence of epithelial cells. cDNA was synthesized using a QuantiTect Reverse Transcription Kit (Qiagen) and 600 ng of RNA template. cDNA samples were then used for qPCR using HOT FIREPol EvaGreen qPCR Supermix (Solis BioDyne). Primers were used at a final concentration of 200 nM (universal forward and reverse for *ACT1*, species-specific forward and reverse for *ECE1*). qPCR amplifications were performed using a Rotor-gene system (Corbett). *ECE1* gene expression was calculated individually for each strain during culture on Caco-2 epithelial cells using the threshold cycle (ΔΔCT) method to calculate fold change (2^ ΔΔCT) against the *ACT1* reference gene. Data shown represent fold change in expression relative to the SC5314 reference control (set as 1). The following oligonucleotide primers 5′-3′) were used to quantify *ECE1* gene expression. *C. albicans* SC5314 (Fw: ctttatcttctcaagctgc, Rev: caacaacagaatcaatatcttc), *C. dubliniensis* CD36 (Fw: gctgatcctgttgttgctgaacc, Rev: atggcatatcagcaatgacaccag), *C. tropicalis* MYA-3404 (Fw: gatgctgtcttagctggttctg, Rev: catctctcttaacaaggccagc), and universal ACT1 (Fw: ccaggtattgctgaacgtatgc, Rev: ggaccagattcgtcgtattcttg).

### Quantification of cellular damage

Caco-2 cells were challenged with fungi at MOI 0.01 for 24 h. A Cytox 96 non-radioactive cytotoxicity assay kit (Promega) was used according to the manufacturer’s instructions. Recombinant porcine lactate dehydrogenase (Sigma) was used to create a standard curve.

### Fungal sequencing

The fungal whole genome sequencing data have been deposited to Sequence Read Archive under BioProject identifier PRJNA1463628. *C. albicans* cells (10 mL) grown overnight on YPD (30 °C, 200 rpm shaking) were harvested by centrifugation at 650 x g for 4 min and the supernatant was removed. The cell pellet was frozen at −20 °C for 30 min before resuspending in 0.5 mL spheroplasting buffer (36.43 g sorbitol plus 10 mL 1 M potassium phosphate buffer (pH 7.0), 4 mL 0.5 M EDTA (pH 7.5) and H_2_O to a volume of 200 mL (filter sterilized). Prior to use, 1% (v/v) 2-mercaptoethanol was added and incubated for 15 min at 37 °C. Spheroplasts were harvested by centrifugation at 750 x g for 4 min and the supernatant discarded. A 1.5mL volume of guanidine solution (21.5 g Guanidine-HCl plus 10 mL 0.5 M EDTA, 0.44 g NaCL, 0.25 mL of 10% sarkosyl and H_2_O to a volume of 50 mL, adjusted to pH 8.0) was added and the pellet gently resuspended. Pellets were incubated at 65 °C for 20 min before cooling on ice. Ethanol (1.5 mL of 100%) was added, samples inverted, and stored at −20°C overnight. Pellets were treated with 0.5 mL RNase solution (10 x TE buffer [pH 8.0] plus 50 μg/mL RNase A [DNase free]) for 1 h at 37 °C. Proteinase-K solution (15 μL of 20 mg/mL in 20 mM CaCl2 10 mM Tris-HCl [pH 7.5]) was then added to the tubes and incubated for a further 1 h at 65 °C. DNA was then extracted by adding 0.5 mL phenol:chloroform:isoamyl alcohol (Sigma- Aldrich, UK), inverting and pulsing in a microcentrifuge. The upper DNA-containing layer was transferred to a fresh tube and phenol:chloroform:isoamyl alcohol extraction was repeated. DNA was precipitated by adding 1/20 volume of 3M sodium acetate (pH 5.2) and 1/20 volume of 100% ethanol, microcentrifuging at full speed, then the ethanol was completely removed. DNA pellets were air-dried for 20 min before resuspending in 15 μL water. Extracted genomic DNA was sequenced using Illumina methodology in the Centre for Genome Enabled Medicine (University of Aberdeen).

### Bioinformatics

Read quality was first assessed using FastQC (v0.12.1) and low-quality bases and adapters were trimmed using TrimGalore (v0.6.5). Reads were then aligned to the reference genome using Bowtie2 (v2.4.5) and alignment metrics were gathered and analysed using Samtools (v1.10). Variants were called using the GATK HaplotypeCaller pipeline. In brief, aligned SAM files were sorted, converted to BAM files and indexed, then the HaplotypeCaller function was called on the processed BAM files. Variants called by the HaplotypeCaller were then hard filtered with the following recommended thresholds; "QD < 2.0", "FS > 60.0", "MQ < 40.0", "SOR > 4.0", "MQRankSum < −12.5", "ReadPosRankSum < −8.0". Base Quality Score Recalibration was then performed (ApplyBQSR) using the filtered variant calls and the previously generated BAM file. Variants were then recalled using the HaplotypeCaller function. Finally, variants called using the recalibrated BAM file were filtered using the same thresholds as above to produce a final VCF file for each sample. VCF files for each sample were merged into a single cohort VCF file using bcftools (v1.19), then indexed and converted to a table using the GATK VariantsToTable function. To produce phylogeny trees, the cohort VCF file generated above was converted to a phylip file using the vcf2phylip package (v2.8) then processed using RAxML (v8.2.12) with GTR, optimization of substitution rates, and GAMMA model of rate heterogeneity (without bootstrapping). Finally, the GTRGAMMA model was used with 100 bootstraps to produce a ‘best tree’.

### Pulsed SILAC proteomics

The mass spectrometry proteomic data have been deposited to the ProteomeXchange Consortium via the PRIDE [1] partner repository under dataset identifier PXD027433 (http://central.proteomexchange.org/?pxid=PXD027433). Selected *C. albicans* strains were cultured overnight in YNB medium (supplemented with sucrose and ammonium sulphate) in either normal oxygen or hypoxic conditions (~98% N_2_, ~ 2% O_2_). The cultures were then switched to pSILAC medium (R and K-free RPMI medium supplemented with K8 and R6 amino acids). Cultures were maintained under these conditions for 3 h before the cells were collected for pSILAC proteomic analysis. Data were input into FungiDB (https://fungidb.org/fungidb/app/) and CGD Gene Ontology Term Finder (http://www.candidagenome.org/cgi-bin/GO/goTermFinder) to identify major biological pathways upregulated under hypoxic or normal oxygen conditions. Enrichment analysis was performed using REVIGO web server (http://revigo.irb.hr/) to generate results for each strain/condition in two-level hierarchical format, from which the most significant gene ontology terms were rendered as pie charts using CirGO software [80].

### *C. albicans* transformations

Transformations were performed as described in Gratacap et al. [53]. Briefly, strains were constructed by transforming CA16 and CA47 with the pENO1-iRFP-NATr plasmid which contains a codon-optimized version of the iRFP gene under the control of constitutive *ENO1* promoter, as well as a nourseothricin resistance (NATr) selection marker (pUC57 backbone; Genscript, Germany). The transformation was carried out using lithium acetate as previously published [81], with nourseothricin resistance as an integration marker (100 μg/mL NAT, Werner Bioagents). Twenty colonies were selected and screened for fluorescence by flow cytometry (488/585 nm, FACScalibur, Becton Dickinson). A PCR check for integration was performed using the following primers to verify correct plasmid integration (1185bp): pENO1 FW: 5’tccttggctggcactgaactcg-3′ and iRFP REV: 5−5’atcacatgaagtcaaatcaacttttctagc-3′. Successful transformation was confirmed via fluorescence microscopy of fungal strains using a ZEISS Imager M2 upright microscope.

### Gut-on-a-chip infection assay

Intestine S1 chips (Emulate, Boston MA) were activated using ER-1 solution and exposed for 20 min under UV light (36 W, 365 nm), then rinsed with ER-2 solution followed by PBS (Gibco) as per the manufacturer instructions. Next, ECM coating solution (100 mg/mL matrigel [Corning] and 30 μg/mL rat tail collagen type 1 [Gibco] diluted in DMEM [Gibco]) was added and incubated for 4 h at 37°C 5% CO_2_. HUVEC cells were seeded at a concentration of 6 x 10^6^/mL in the bottom channel and the chips inverted for 2 h at 37°C 5% CO_2_. After cell attachment, chips were returned to upright and washed with HUVEC medium supplemented with 1% Penicillin-Streptomycin and 1% L-glutamine. Caco-2 cells were then seeded into the bottom channel at a concentration of 3 x 10^6^/mL and incubated for 2 h. Chips were gravity washed before remaining static overnight. Finally, chips were connected to the Emulate platform and maintained for the duration of culture using the following conditions: 37 °C, 5% CO_2,_ 30 μL/h flow, 10% lateral mechanical stretching at a frequency of 0.15 Hz. Cell culture medium was equilibrated and refreshed every 3 d.

At the start of the experiment timeline, the inlet reservoir for the top channel was filled with Caco-2 culture medium containing a known concentration of fungi (8 x 10^4^/mL). This was then flowed through the chip for 30 min at 400 µL/h via the top channel, while 30 µL/h sterile medium was delivered via the bottom channel to synchronize the medium. The chip was then imaged for t = 0 time point to visualise fungi before incubating for a further 30 min at 30 µL/h with flow and stretch applied (10% and 0.15 Hz). The channels were then washed through with complete medium supplied via the inlet ports at 400 µL/h with no flow or stretch applied. Each channel was washed a further three times with PBS before the channels were fixed with 4% PFA for 20 min. After incubation, the channels were washed with PBS x 3 before filling and submerging in PBS for storage at 4 °C until subsequent imaging.

### Gut-on-a-chip immunofluorescence

Organ chip channels were washed with PBS then stained to visualise hyphal invasion and structural features. Each channel was filled with 100 µL blocking buffer and tips filled with buffer were left in the ports. Chips were then incubated either overnight at 4 °C or for 2 h at room temperature in a humidified box. Tips were removed from ports and channels were washed three times with PBS. Primary antibodies were prepared in dilution buffer and 100 µL was added to each channel before leaving overnight at 4 °C in a humidified box. Primary antibodies were 1:200 rabbit anti-ZO-1 (Abcam, EPR19945-296) and 1:200 mouse anti-E-cadherin (Abcam, HECD-1). After incubation, both channels were washed three times with PBS before 100 µL secondary antibody mix was added and the chips left to incubate for 2 h in a humidified box at room temperature in the dark. Secondary antibodies were 1:500 AF488 phalloidin, 1:500 AF568 goat anti-rabbit, 1:500 AF647 donkey anti-mouse (ThermoFisher) prepared in dilution buffer. After incubation, both channels were washed with PBS three times. Chips were submerged in PBS until imaging, at which point they were mounted onto clear coverslips with ProlongFade and PBS. Imaging was performed using an inverted Zeiss 880 Laser Scanning Confocal Microscope with AiryScan and analysis was conducted on ImageJ (Version 2.3.0).

### Statistics

Statistical analyses were conducted using Prism 9 software (GraphPad, USA). Data normality was assessed using Anderson-Darling, D’Agostino and Pearson, Shapiro-Wilk, and Kolmogorov-Smirnov tests as indicated. For unpaired data, Mann Whitney test was used to compare two groups assuming unequal variance, while one-way ANOVA with a Kruskal-Wallis post-hoc test was employed for comparisons involving more than two groups. A paired *t* test was used for two-group comparisons, and a repeated measures two-way ANOVA with Bonferroni’s multiple comparisons post-hoc test was applied for comparisons involving more than two groups. Statistical significance was defined as a p-value of <0.05.

## Supporting information

S1 Fig Flow-cytometry gating strategy for T-cell identification and isolation. Peripheral blood Vγ9Vδ2+ T-cells were identified as viable singlet lymphocytes co-expressing CD3 and Vδ2. For γδ T-APC assays, the Vγ9+ population was magnetically enriched to >99% purity prior to APC generation (see methods), bulk CD4^+^ T cells were isolated from PBMC via negative selection (>95% purity), and allogeneic naïve CD4+ T-cell responders were sort-purified as CD3+ CD4+ lymphocytes that co-expressed CCR7 and CD45RA (>99% purity). On completion of mixed leukocyte co-cultures, cytokine expression in the stimulated CD4+ T-cells was gated relative to fluorescence minus one controls.
(TIF)

S2 Fig Phylogeny of human gut-adapted *C. albicans* strains. A phylogenetic tree of *C. albicans* clinical isolates was generated based on variant calling in the gut-adapted strains integrated with genomic sequences from n = 182 samples in the Pasteur collection [83]. Variant data for each sample were merged into a single VCF file using bcftools (v1.19), then indexed and converted into a table using the GATK VariantsToTable function. To produce phylogeny trees, the cohort VCF file was converted using vcf2phylip (v2.8) and then processed by RAxML (v8.2.12). The data underlying this Figure can be found in https://doi.org/10.5281/zenodo.23063566.
(TIF)

S3 Fig Hypoxia-induced protein synthesis in gut-adapted *C. albicans* strains. Volcano plots show newly synthesized proteins that were upregulated or downregulated in CA16 versus CA47 under hypoxic conditions (Hx) or during normal oxygen culture (Nx). Proteins with positive log2 fold change and significant p-values represent candidates induced by hypoxia. Venn diagram shows minimal overlap in hypoxia-induced proteins synthesized by CA16 and CA47. Heat-map displays proteins most strongly induced by hypoxia in strain CA16. The data underlying this Figure can be found in https://doi.org/10.5281/zenodo.23064305.
(TIF)

S4 Fig Strains CA16 and CA47 exhibit distinct proteomic responses to hypoxia. The pSILAC data were interrogated to identify GO terms for fungal proteins that were >50% differentially expressed between conditions. Enrichment analysis was performed using REVIGO web server (http://revigo.irb.hr/) to generate results for each strain/condition in two-level hierarchical format, from which the most significant gene ontology terms were rendered as pie charts using CirGO software [80].
(TIF)

S5 Fig Impaired NETosis response to intestinal *C. albicans* in Crohn’s disease. Blood neutrophil NETosis response to filamentous gut strain CA16 was significantly impaired in patients with CD relative to healthy donors (A), whereas macrophage IL-1β release was comparable between cohorts across a range of fungal stimuli (B). Differences between groups were assessed by 2-way ANOVA (**p < 0.01). The data underlying this Figure can be found in https://doi.org/10.5281/zenodo.23064172.
(TIF)

S6 Fig Hyphal strain CA16 is suppressed by Vδ2+ T-cells in healthy gut tissue. Restricted growth of CD-derived strain CA16 after spiking into healthy colonic biopsy cultures that were stimulated or not with the selective Vδ2+ cell agonist HMB-PP (10nM) for 3 days at 37 °C. Differences in fungal burden between wells were assessed by paired *T* test (*p < 0.05). The data underlying this Figure can be found in https://doi.org/10.5281/zenodo.23064172.
(TIF)

S1 Table Donor demographics, clinical data, and fungal isolation rates. Demographic details of the control donors (n = 28) and CD patients (n = 43) who provided blood and tissue samples used in this study. Data shown include Montreal classification, current therapy, and treatment history where available, as well as human / fungal sample distribution across the figures. Rates of fungal species isolation from mucosal tissue cultures in each cohort are also shown. Both groups included equal numbers of male and female donors (1:1), while the control cohort (mean age 46; range 21–80) was significantly older than the CD cohort (mean age 31; range 16–60) reflecting typical demographics of the gastroenterology clinic (p = 0.0005 by Welch’s *t* test).
(XLSX)

S2 Table Proteins identified by pSILAC analysis of gut-adapted strains. *C. albicans* strains were cultured overnight in YNB medium (supplemented with sucrose and ammonium sulphate) in either normal oxygen or hypoxic conditions (~98% N_2_, ~ 2% O_2_). The cultures were then switched to pSILAC medium (R and K-free RPMI medium supplemented with K8 and R6 amino acids). Cultures were maintained under these conditions for 3 h before the cells were collected for pSILAC proteomic analysis. The mass spectrometry proteomic data were deposited in the ProteomeXchange Consortium via the PRIDE partner repository (dataset identifier PXD027433).
(XLSX)

S1 Video Fungal adhesion in a dynamic human ‘gut-on-a-chip’ model. Video moves from vascular channel up through the z-axis of the model tissues to visualize epithelial surface binding of strains CA16 and CA47 (fluorescent red) at 2 hours post infection. *C. albicans* was added at a concentration of 8 x 10^4^ /mL cells and allowed to adhere for 30 mins before non-adherent cells were washed away. (Zeiss 880 Laser Scanning Confocal Microscope).
(MP4)

S2 Video Vascular invasion of strain CA16 in a human ‘gut-on-a-chip’ model. Video moves through the vascular channel, revealing CA16 hyphal protrusions from the epithelial channel at 24 hours post infection (x20 magnification). CA16 was added at a concentration of 8 x 10^4^ /mL cells and allowed to adhere for 30 mins before non-adherent cells were washed away. (Zeiss 880 Laser Scanning Confocal Microscope).
(MP4)

## Abbreviations

CD: Crohn’s disease
CLYS: candidalysin
HMB-PP: (*E*)-4-Hydroxy-3-methyl-but-2-enyl pyrophosphate
IBD: inflammatory bowel disease
NATr: nourseothricin resistance
NLS: NET-like structures
Vδ2+: Vγ9Vδ2+
