# Supplementary material for: Fungal suppression by bacterial metabolite-activated human intestinal γδT-cells is disrupted in Crohn’s disease
Source: PLoS Biol. 2026 Nov 2;24(11):e3004080. doi: 10.1371/journal.pbio.3004080 (PMC13750660; doi:10.1371/journal.pbio.3004080)
Supplement: Supplementary file 1 [file pbio.3004080.s013.doc]

**Reviewer #1**

The authors demonstrate a decrease in activated Vδ2 T cells fails to induce an IL-22 response to suppress the outgrowth of Candida albicans with pathogenic features. Both the number of Vδ2 T cells and their responsiveness to HMB-PP was compromised in CD. Expansion of these Candida isolates resulted in increased epithelial adhesion and invasion. Although macrophage IL-1b expression was not compromised, neutrophil NETosis was diminished in those with CD. Although this manuscript does not represent a substantial advance in the field, this study nicely builds upon previous findings and links Vδ2 T cell induction of IL-22 to fungal suppression while also investigating the effects of a CD-derived Candida isolate on the epithelial barrier and anti-fungal immunity. Further, the models generated, including human gut-on-a-chip, will be valuable for future investigation.

**1.**	It is not clear which fungi are shown in Fig 1G. There is no description of a gating strategy or how the flow cytometry was performed for fungi. There are no corresponding symbols provided in the figure legend for Fig 1H. This information would provide greater clarity for the conclusions drawn.

*Apologies for omitting these details: Fig 1G shows growth / suppression of biopsy-derived Trichoderma viride under the treatment conditions indicated (now specified in the legend). We have also added a full description of the flow-cytometry and fungal gating / quantification strategy in the methods section. The symbols representing different fungal species in Fig 1H are now identified in a key and explained in the legend.*

**2.**	Similar to the point above, a gating strategy should be provided for Fig 2E. Were all Vδ2 T cells assessed ones that egressed from the culture? Are there Vδ2 T cells that remain in the tissue? Does Fig 2E show Vδ2 from active or inactive CD?

*A flow-cytometry gating strategy for identifying / sorting Vγ9δ2+ lymphocytes and naïve CD4+ T-cell responders is now shown in Supplementary Figure 1, and an example cytokine expression analysis is displayed in new Fig 2G (full details are also provided in our previous manuscript, PMID:28330898). Fig 2E shows peripheral blood Vδ2 T cells after culture with or without exogenous HMB-PP (rather than biopsy-derived Vδ2 T cells which were not numerous enough to facilitate this type of assay). In this context, there is no mucosal tissue to potentially retain the Vδ2+ cells. Fig 2E shows example data from a patient with inactive CD (now clarified in the legend text).*

**3.**	In Fig 6A, it would be helpful to show an inset of a higher magnification image of the NLS for it to be more easily appreciated. For both NLS and IL-1b production, it would be more informative to compare the response between cells isolated from those with HC and CD, this would better highlight the defective CD neutrophil response to CD16 (two-way ANOVA).

*A higher-magnification image of the NET-like structures has now been added to Fig 6A. We also performed the suggested 2-way ANOVA for both the neutrophil data and monocyte-macrophage assays, which did indeed confirm a defective CD neutrophil response to CA16 (p=0.0057), consistent with earlier reports of IBD-linked functional impairment in this compartment (PMID:2831119). These data have been added in Supplementary Fig 5.*

**4.**	In Fig 7D,E, it should be indicated in the figure legend that these are images of the epithelium.

*The Fig 7D,E panels have now been labelled and the legend updated accordingly.*

**Reviewer #2**

This study investigates the interaction of host immunity and fungal commensals in Crohn's disease (CD) to understand the impact of these on disease progression. This is an important question to address as most studies have mainly focused on the role of bacterial commensals in CD, which means are understanding on the role of fungi in CD progression is limited. In this study, the authors initially focus on how bacterial metabolic activation of Vγ9Vδ2+ T-cells impacts fungi (particularly Candida albicans) in intestinal cultures. They next investigate C. albicans isolates from CD patients and find they appear to have enhanced capability to activate innate immunity and invade epithelial barrier (in a gut-on-a-chip) model. This work utilises a wide array of techniques and focus on using primary human samples and patient originated fungal isolates.

**1.**	This manuscript has the title "Fungal suppression by human intestinal γδ T-cells is disrupted in Crohn's disease", suggesting the focus will be how γδ T cells directly suppress fungi. Indeed, the opening 2 figures (Figs. 1 & 2) show with intestinal cultures from biospies that CD patients have more outgrowth of fungi and less activated γδ T-cells. Yet, following this, (Figs 3 - 7) the focus switches to C. albicans isolates from CD patients and how one in particular (CA16) is more stimulatory of innate cells and able to invade epithelial cells in a gut on a chip model. However, the authors didn't directly test whether CA16 is still suppressed by γδ T cells (or if this is directly impaired in γδ T cells from HC vs CD patients). Therefore, could the isolates once emerged become more resistant to γδ t cells? Therefore, have the authors performed any experiments to determine to test whether the highlighted CA16 strain highlighted is still susceptible to γδ T cell-mediated suppression?

Thank you for this excellent suggestion which we have addressed in a new series of fungal suppression assays using colonic biopsy samples from healthy control donors (n=5). Following the same protocol as before, tissue explant cultures were stimulated or not with HMB-PP before spiking in *C. albicans* isolate CA16 and quantifying fungal growth by flow-cytometry on day 3. These assays confirmed that hyphal strain CA16 is significantly suppressed by Vδ2+ T-cell activation in healthy mucosal tissue (p=0.030; Supplementary Fig 6), consistent with our earlier finding that Vδ2+ T-cells can also restrain the growth of ‘hyper-virulent’ reference strain SC5314 (Fig 2C) (which is similarly filamentous and induced comparable levels of monocyte-macrophage damage in our immunoassays; Fig 6C). These new data confirm that the more pathogenic features of strain CA16 do not overcome fungal suppression when intestinal Vδ2+ T-cell numbers and function are intact.

In contrast, CD mucosal tissue contains only trace numbers of Vδ2+ cells that are highly refractory to stimulation (Fig 2A,B), rapidly overgrown by fungi (Fig 2C,D), and thus quickly lost to follow-up analyses. As a result, it is very challenging to perform direct functional experiments using the γδ T-cell fraction of IBD biopsy samples (as noted by another recent high-profile study, PMID: 41650251). Our current data do however show that intestinal Vδ2+ cells from CD patients are poorly responsive to HMB-PP *ex vivo* (Fig. 2B), and by using blood-based assays to obtain sufficient cells, we confirmed that Vδ2-mediated IL-22 responses are indeed impaired in active CD (Fig. 2E-H). We were further able to verify that IL-22 is the critical factor required for Vδ2-mediated fungal suppression in human gut biopsy cultures (Fig. 1G,H). Our report therefore demonstrates that poor fungal control in CD is linked with defects in Vδ2 reactivity and functionality, as well as low cell numbers in mucosal tissue. These findings align with our previous report which analysed individual mucosal Vδ2 T cells by flow cytometry, revealing CD-linked changes in HMB-PP reactivity and increased expression of Th1 cytokines including IFNγ (PMID: 26168223). Importantly, IFNγ is known to suppress activity of RORγt which is the key transcriptional driver of IL-22 expression (PMID: 34022896), consistent with a reduced ability of Vδ2+ cells to produce / induce IL-22 in CD (PMID: 33771991). We do however acknowledge that we cannot resolve whether loss of fungal control in CD is due mainly to functional impairment or numerical deficits in the Vδ2+ T-cell population (although our data clearly show that both features are present). We have therefore updated the discussion to reflect this limitation (p.15).

**2.**	The data in Fig. 1 and Fig. 2 focus on Vδ2+ γδ T cells (activated via HMB-PP) and their role in limiting intestinal cultures.  The use of cultures from human biopsies is commendable and ensures the data is highly relevant to processes within intestines. However, most of the data shown in Fig. 1A -F are representative images of outgrowth of bacteria and fungi. Have the authors performed assays to quantify these data and show how consistent this is across donors?

Our thanks to the reviewer for recognising the importance of working with human gut tissue despite the technical challenges this entails! In the CD group, we obtained 6 biopsy-derived fungal isolates from a total of n=15 tissue donors, meaning that 40% of explant cultures yielded a viable gut-adapted species or strain. In the control group, we obtained 11 fungal isolates from a total of 20 tissue donors, with two of the gut-adapted species being obtained from an individual patient CA31 (*C. albicans* and *C. glabrata*). This equates to 50% of control cultures displaying spontaneous fungal outgrowth, which was not significantly different to rates observed in the CD cohort (p=NS; Fisher exact test). These data have been appended to Supplementary Table 1 (columns N-R).

The authors go onto to show representative flow cytometry plots to highlight how blockade of αIL-22 increased fungal filamentation (top right gate) yet they don't present this data in a graph to show consistent across donors.

This interesting filamentation effect was seen only with mold species *Trichoderma viride* (Fig 1G), so we have displayed these data but avoided making broader claims for the observation (now clarified in the legend text).
It was not possible to differentiate *C. albicans* growth patterns in the same way (e.g. Fig 2A).

They do present a graph of fungal burden, but the different symbols are not explained. Neither is data shown on this graph about the impact of αIFNγ (but representative flow plots are shown).

Apologies for this omission - the symbols indicating different fungal species are now shown in the Fig 1H key and explained in the legend text. A summary graph of the αIFNγ data has also been included in Fig 1H.

Data in Fig 2 shows clear differences in the number of Vδ2+ γδ T cells in HC vs CD, showing that numbers are reduced in CD (accompanied with a greater fungal burden). To address if this γδ T cells from CD are impaired (as well being reduced in number) flow cytometry analysis is undertaken from cells isolated from the blood and stimulated with HMB-PP. Were these blood samples from the same individuals that provide intestinal samples?

Due to logistical constraints the blood samples were collected from a separate patient cohort (detailed in Supplementary Table 1 and now clarified in the Fig 2. legend text).

However, the presentation of these data is confusing. Flow plots of unstimulated vs HMB-PP plots are shown (Fig. 2E), but it is unclear which of these are shown in graphs presented in Fig. 2F. Some markers are shown on plots but not quantified on the graphs (e.g. ICOS-L).

For improved clarity, these graphs have now been updated to show all the phenotypic markers assessed, including ICOS-L and CD70 (which were not significantly different between groups; Fig 2F).

Whereas the percentage of HLA-DR+ Vδ2+ is shown on graphs but no gate is shown on the plots to show how that data was achieved. This should be amended so that presentation of the flow plots and graphs are clearer.

The HLA-DR gate was set relative to a fluorescence minus one control and is displayed in the first column of Fig 2E. An example flow-cytometry gating strategy has also been added in new Supplementary Figure 1.

Furthermore, in Fig 2G I assume that CD4+ T cells expression of IL-22 was after culture with Vδ2+ γδ T cells? Can the authors show representative plots to confirm the staining of these populations?

Yes - previous Fig.2G showed CD4+ T-cell expression of IL-22 induced by co-culture with Vδ2+ cells. This result is now displayed in Fig.2H together with a representative plot of the CD4+ T-cell cytokine analysis (new Fig.2G).

**3.**	The authors go onto show the diversity of C. albicans strains from colonic tissue (Fig. 3). It is unclear how many robust the data generated in A and B due to a lack of quantification. Have the authors undertaken any other approaches (e.g. sequencing) to confirm in an unbiased manner that C. albicans is the dominant sp to other fungi in the tissue?

As described in our answer to comment #2 above, 40-50% of biopsy explant cultures yielded one or more viable fungal isolates, and this was not significantly different between cohorts (now shown in Supplementary Table 1). This is consistent with a previous study of viable human gut fungi in which *C. albicans* was by far the dominant species, being isolated from 39.8% of n=111 healthy volunteers (PMID:27536299). We did not employ sequencing approaches as these detect non-viable fungi / dietary contaminants that are only transiently present and do not represent true gut commensals (PMID:29178920), while also offering no opportunity to conduct functional immunoassays such as those described in our report. Importantly, while the fungal composition of human stool differs significantly from analyses of mucosal tissue (PMID:26574491), and culture-dependent and culture-independent methods yield distinct mycobiome profiles (PMID:28824566), in each case these methods routinely identify Candida species as dominant / co-dominant in the human gut, especially in patients with IBD.

Rather than replicate many previous gut microbiota profiling / sequencing studies, our culture-based method aimed to isolate viable fungal species for use in functional immunoassays. For this, we used RPMI-1640 chemically defined growth medium, which is widely used for culturing fungi and is the standard base media for anti-fungal susceptibility testing protocols established by CLSI and EUCAST. Accordingly, our method was able to support the outgrowth of multiple different yeasts and moulds (Fig. 1), among which Candida was the most prevalent genus and *C. albicans* the most dominant species - aligning closely with the published literature.

Sequencing of the Candida isolates show little changes at the genomic level (Fig. 4) but that growing one of the stains (CA16) in hypoxic conditions were more filamentous compared to isolates from a healthy patient (Fig. 5). This is fascinating data, but could the authors give slightly more rationale on why they only focused on this one isolate? Did they test the other isolates they found and see different growth patterns in hypoxic conditions?

We are pleased that the reviewer found these data interesting! Since *C. albicans* phenotype was largely consistent between isolates within each patient group (Fig 3), we elected to deeply characterise one exemplar strain per cohort rather than conducting a shallower analysis of the entire collection. For this, we selected one strain with typical characteristics of the CD group (CA16) and one representative of the HC group (CA47), since these displayed ‘opposite’ filamentation phenotypes *in vitro* (Fig 3E). These strains were also closely matched for donor age, sex, and minimal complicating treatments, hence any differences between them would more likely reflect true fungal biology rather than an impact of host variables. This rationale has now been explained more clearly in the revised text (p.9). We thoroughly compared and contrasted these exemplar strains under different environmental conditions (Fig 5), via robust testing in multiple types of immunoassay (Fig 6), and assessed tissue invasion capacity in our custom gut-on-a-chip model (Fig 7), thereby generating a comprehensive picture of how divergent *C. albicans* phenotypes can impact human host biology. While it would certainly be interesting to pursue, undertaking the full range of these analyses for our entire library of *C. albicans* isolates would be a major task, highly expensive, and very time consuming (hence this will be a long-term endeavour).

Using pSILAC proteomics show a table of hits and present 4 example proteins identified. It would be of interest if the proteomics data could be more integrated with further analysis (e.g. Venn Diagrams) so the reader can ascertain how many proteins were altered/unaltered between the two strains in hypoxic vs normal conditions.
Thank you for this useful suggestion - we have now added a series of Volcano plots together with a heat-map analysis and Venn diagram to better illustrate how hypoxia modifies the proteome differentially in strains CA16 and CD47 (new Supplementary Figure 3).

The authors go on to show that CA16 activates elevated neutrophil and macrophages suggesting that CD isolates are more inflammatory (Fig. 6). Yet, the reason for this isn't explored. Have the authors tested whether if this is due to CA16 having greater exposure of key fungal PAMPs like β-glucan (or that C. albicans from HC have lower exposure)?

Our hypothesis was that the pro-inflammatory effects of CD isolates are due to hyphal induction of NETosis and physical injury to macrophages caused by filamentation and toxin production, as well as inflammasome triggering / IL-1β release (PMID:33876755, 36130496), all of which are assessed in our report. We do however agree that differential exposure of fungal PAMPs may also play a role. We therefore assessed strain-dependent variation in levels of chitin, mannan, and β-glucan exposure between *C. albicans* isolates from each cohort. These analyses did indeed confirm that β-glucan levels were increased among CD strains (p=0.0317), consistent with their more pronounced hyphal morphology, but no other significant differences were detected between groups. These data have now been added to updated Fig 3C.

**4.**	The final figure utilises gut-on-a-chip to reveal that CA16 is more able to adhere and invade the intestinal barrier. The authors highlight this system does not have immune cells (e.g. γδ T cells) so they can't test if they help prevent this process. However, this is a very useful system that enables the authors to make some interesting observations about these isolates in a physiologically relevant setting. Have the authors attempted to alter the epithelium within these chips (e.g. to seed with epithelium from CD patients) to see how this might shape C. albicans behaviour and explain how CA16 originated? E.g. could CA47 from a HC become invasive if in a CD like gut environment?

These are excellent suggestions and we will certainly attempt these types of experiment in future work! It would be fascinating to introduce patient-derived epithelium and immune cells into the gut chip model and assess interactions with different *C. albicans* strains and phenotypes, but these assays are highly complex and will require extensive optimisation to achieve (a current PhD student is developing bioengineering solutions to explore these ideas, but this will be a long-term project and thus beyond scope of the current report).
