# Supplementary material for: Fungal suppression by bacterial metabolite-activated human intestinal γδT-cells is disrupted in Crohn’s disease
Source: PLoS Biol. 2026 Nov 2;24(11):e3004080. doi: 10.1371/journal.pbio.3004080 (PMC13750660; doi:10.1371/journal.pbio.3004080)
Supplement: Peer review history

# Editor report: Decision Letter 0

**DOI:** 10.1371/journal.pbio.3004080.r001

**Authors:** Melissa Vazquez Hernandez

29 Mar 2026

Dear Dr McCarthy,

Thank you for submitting your revised manuscript entitled "Fungal suppression by human intestinal γδ T-cells is disrupted in Crohn’s disease" for consideration as a Research Article by PLOS Biology.

Your manuscript has now been evaluated by the PLOS Biology editorial staff as well as by an academic editor with relevant expertise and I am writing to let you know that we would like to send your submission out for external peer review.

However, before we can send your manuscript to reviewers, we need you to complete your submission by providing the metadata that is required for full assessment. To this end, please login to Editorial Manager where you will find the paper in the 'Submissions Needing Revisions' folder on your homepage. Please click 'Revise Submission' from the Action Links and complete all additional questions in the submission questionnaire.

Once your full submission is complete, your paper will undergo a series of checks in preparation for peer review. After your manuscript has passed the checks it will be sent out for review. To provide the metadata for your submission, please Login to Editorial Manager (https://www.editorialmanager.com/pbiology) within two working days, i.e. by Mar 31 2026 11:59PM.

If your manuscript has been previously peer-reviewed at another journal, PLOS Biology is willing to work with those reviews in order to avoid re-starting the process. Submission of the previous reviews is entirely optional and our ability to use them effectively will depend on the willingness of the previous journal to confirm the content of the reports and share the reviewer identities. Please note that we reserve the right to invite additional reviewers if we consider that additional/independent reviewers are needed, although we aim to avoid this as far as possible. In our experience, working with previous reviews does save time.

If you would like us to consider previous reviewer reports, please edit your cover letter to let us know and include the name of the journal where the work was previously considered and the manuscript ID it was given. In addition, please upload a response to the reviews as a 'Prior Peer Review' file type, which should include the reports in full and a point-by-point reply detailing how you have or plan to address the reviewers' concerns.

During the process of completing your manuscript submission, you will be invited to opt-in to posting your pre-review manuscript as a bioRxiv preprint. Visit http://journals.plos.org/plosbiology/s/preprints for full details. If you consent to posting your current manuscript as a preprint, please upload a single Preprint PDF.

Feel free to email us at plosbiology@plos.org if you have any queries relating to your submission.

Kind regards,

Melissa

Melissa Vazquez Hernandez, Ph.D.

Associate Editor

PLOS Biology

mvazquezhernandez@plos.org

# Peer review: Decision Letter 1

**DOI:** 10.1371/journal.pbio.3004080.r002

**Authors:** Melissa Vazquez Hernandez

28 May 2026

Dear Neil,

Thank you so much for your patience while your manuscript "Fungal suppression by human intestinal γδ T-cells is disrupted in Crohn’s disease" was peer-reviewed at PLOS Biology. It has now been evaluated by the PLOS Biology editors, an Academic Editor with relevant expertise, and by two independent reviewers.

In light of the reviews, which you will find at the end of this email, we would like to invite you to revise the manuscript to thoroughly address the reviewers’ reports. Both reviewers are positive about the relevance of the question, the use of primary human tissue and patient-derived Candida isolates, and the potential value of the human gut-on-a-chip model. However, several important issues will need to be addressed before the manuscript can be further considered. Most importantly, Reviewer #2 raises a central concern about the connection between the two main parts of the study: while the first figures support a role for Vδ2 T cell/IL-22-mediated suppression of fungal outgrowth, the later figures focus on the pathogenic features of the CD-derived C. albicans isolate CA16 without directly testing whether this strain remains susceptible to Vδ2 T cell-mediated suppression, or whether suppression differs between Vδ2 T cells from healthy controls and CD patients. The reviewers also request clearer and more quantitative presentation of the fungal outgrowth and flow cytometry data, including gating strategies, explanation of symbols, quantification across donors, and clarification of whether blood and biopsy samples came from the same individuals. In addition, the revision should better justify the focus on the CA16 and CA47 isolates, clarify the robustness of the fungal diversity analysis and whether unbiased approaches were used to confirm C. albicans dominance, and provide deeper interpretation of the proteomics data and possible mechanisms underlying the increased inflammatory activity of CA16, such as altered fungal PAMP exposure. Finally, the reviewers ask for several improvements to figure presentation and interpretation, including clearer labeling of epithelial images in the gut-on-chip experiments, higher-magnification visualization of NET-like structures, and more direct comparison of HC versus CD innate immune responses. We agree that these points are important and consider that additional analyses, clarification, and where feasible further experiments addressing these concerns would substantially strengthen the manuscript.

Given the extent of revision needed, we cannot make a decision about publication until we have seen the revised manuscript and your response to the reviewers' comments. Your revised manuscript is likely to be sent for further evaluation by all or a subset of the reviewers.

In addition to these revisions, you will need to complete some formatting changes, which you will receive in a follow up email. A member of our team will be in touch with a set of requests shortly.

We expect to receive your revised manuscript within 3 months. Please email us (plosbiology@plos.org) if you have any questions or concerns, or would like to request an extension.

At this stage, your manuscript remains formally under active consideration at our journal; please notify us by email if you do not intend to submit a revision so that we may withdraw it.

**IMPORTANT - SUBMITTING YOUR REVISION**

Your revisions should address the specific points made by each reviewer. Please submit the following files along with your revised manuscript:

1. A 'Response to Reviewers' file - this should detail your responses to the editorial requests, present a point-by-point response to all of the reviewers' comments, and indicate the changes made to the manuscript.

*NOTE: In your point-by-point response to the reviewers, please provide the full context of each review. Do not selectively quote paragraphs or sentences to reply to. The entire set of reviewer comments should be present in full and each specific point should be responded to individually, point by point.

You should also cite any additional relevant literature that has been published since the original submission and mention any additional citations in your response.

2. In addition to a clean copy of the manuscript, please also upload a 'track-changes' version of your manuscript that specifies the edits made. This should be uploaded as a "Revised Article with Changes Highlighted" file type.

*Re-submission Checklist*

When you are ready to resubmit your revised manuscript, please refer to this re-submission checklist: https://plos.io/Biology_Checklist

To submit a revised version of your manuscript, please go to https://www.editorialmanager.com/pbiology/ and log in as an Author. Click the link labelled 'Submissions Needing Revision' where you will find your submission record.

Please make sure to read the following important policies and guidelines while preparing your revision:

*Published Peer Review*

Please note while forming your response, if your article is accepted, you may have the opportunity to make the peer review history publicly available. The record will include editor decision letters (with reviews) and your responses to reviewer comments. If eligible, we will contact you to opt in or out. Please see here for more details:

https://blogs.plos.org/plos/2019/05/plos-journals-now-open-for-published-peer-review/

*PLOS Data Policy*

Please note that as a condition of publication PLOS' data policy (http://journals.plos.org/plosbiology/s/data-availability) requires that you make available all data used to draw the conclusions arrived at in your manuscript. If you have not already done so, you must include any data used in your manuscript either in appropriate repositories, within the body of the manuscript, or as supporting information (N.B. this includes any numerical values that were used to generate graphs, histograms etc.). For an example see here: http://www.plosbiology.org/article/info%3Adoi%2F10.1371%2Fjournal.pbio.1001908#s5

*Blot and Gel Data Policy*

We require the original, uncropped and minimally adjusted images supporting all blot and gel results reported in an article's figures or Supporting Information files. We will require these files before a manuscript can be accepted so please prepare them now, if you have not already uploaded them. Please carefully read our guidelines for how to prepare and upload this data: https://journals.plos.org/plosbiology/s/figures#loc-blot-and-gel-reporting-requirements

*Protocols deposition*

To enhance the reproducibility of your results, we recommend that if applicable you deposit your laboratory protocols in protocols.io, where a protocol can be assigned its own identifier (DOI) such that it can be cited independently in the future. Additionally, PLOS ONE offers an option for publishing peer-reviewed Lab Protocol articles, which describe protocols hosted on protocols.io. Read more information on sharing protocols at https://plos.org/protocols?utm_medium=editorial-email&utm_source=authorletters&utm_campaign=protocols

Thank you again for your submission to our journal. We hope that our editorial process has been constructive thus far, and we welcome your feedback at any time. Please don't hesitate to contact us if you have any questions or comments.

Sincerely,

Melissa

Melissa Vazquez Hernandez, Ph.D.

Associate Editor

PLOS Biology

mvazquezhernandez@plos.org

------------------------------------

REVIEWERS' COMMENTS:

------------------------------------

Reviewer #1:

Summary: The authors demonstrate a decrease in activated Vδ2 T cells fails to induce an IL-22 response to suppress the outgrowth of Candida albicans with pathogenic features. Both the number of Vδ2 T cells and their responsiveness to HMB-PP was compromised in CD. Expansion of these Candida isolates resulted in increased epithelial adhesion and invasion. Although macrophage IL-1b expression was not compromised, neutrophil NETosis was diminished in those with CD. Although this manuscript does not represent a substantial advance in the field, this study nicely builds upon previous findings and links Vδ2 T cell induction of IL-22 to fungal suppression while also investigating the effects of a CD-derived Candida isolate on the epithelial barrier and anti-fungal immunity. Further, the models generated, including human gut-on-a-chip, will be valuable for future investigation.

Comments:

1. It is not clear which fungi are shown in Fig 1G. There is no description of a gating strategy or how the flow cytometry was performed for fungi. There are no corresponding symbols provided in the figure legend for Fig 1H. This information would provide greater clarity for the conclusions drawn.

2. Similar to the point above, a gating strategy should be provided for Fig 2E. Were all Vδ2 T cells assessed ones that egressed from the culture? Are there Vδ2 T cells that remain in the tissue? Does Fig 2E show Vδ2 from active or inactive CD?

3. In Fig 6A, it would be helpful to show an inset of a higher magnification image of the NLS for it to be more easily appreciated. For both NLS and IL-1b production, it would be more informative to compare the response between cells isolated from those with HC and CD, this would better highlight the defective CD neutrophil response to CD16 (two-way ANOVA).

4. In Fig 7D,E, it should be indicated in the figure legend that these are images of the epithelium.

------------------------------------

Reviewer #2:

This study investigates the interaction of host immunity and fungal commensals in Crohn's disease (CD) to understand the impact of these on disease progression. This is an important question to address as most studies have mainly focused on the role of bacterial commensals in CD, which means are understanding on the role of fungi in CD progression is limited. In this study, the authors initially focus on how bacterial metabolic activation of Vγ9Vδ2+ T-cells impacts fungi (particularly Candida albicans) in intestinal cultures. They next investigate C. albicans isolates from CD patients and find they appear to have enhanced capability to activate innate immunity and invade epithelial barrier (in a gut-on-a-chip) model. This work utilises a wide array of techniques and focus on using primary human samples and patient originated fungal isolates. I have the following questions for the authors about this work:

1)This manuscript has the title "Fungal suppression by human intestinal γδ T-cells is disrupted in Crohn's disease", suggesting the focus will be how γδ T cells directly suppress fungi. Indeed, the opening 2 figures (Figs. 1 & 2) show with intestinal cultures from biospies that CD patients have more outgrowth of fungi and less activated γδ T-cells. Yet, following this, (Figs 3 - 7) the focus switches to C. albicans isolates from CD patients and how one in particular (CA16) is more stimulatory of innate cells and able to invade epithelial cells in a gut on a chip model. However, the authors didn't directly test whether CA16 is still suppressed by γδ T cells (or if this is directly impaired in γδ T cells from HC vs CD patients). Therefire, could the isolates once emerged become more resistant to γδ t cells? Therefore, have the authors performed any experiments to determine to test whether the highlighted CA16 strain highlighted is still susceptible to γδ T cells mediated suppression?

2)The data in Fig. 1 and Fig. 2 focus on Vδ2+ γδ T cells (activated via HMB-PP) and their role in limiting intestinal cultures. The use of cultures from human biopsies is commendable and ensures the data is highly relevant to processes within intestines. However, most of the data shown in Fig. 1A -F are representative images of outgrowth of bacteria and fungi. Have the authors performed assays to quantify these data and show how consistent this is across donors? The authors go onto to show representative flow cytometry plots to highlight how blockade of αIL-22 increased fungal filamentation (top right gate) yet they don't present this data in a graph to show its consistent across donors. They do present a graph of fungal burden, but the different symbols are not explained. Neither is data shown on this graph about the impact of αIFNγ (but representative flow plots from this are shown). Data in Fig 2 shows clear differences in the number of Vδ2+ γδ T cells in HC vs CD, showing that numbers are reduced in CD (accompanied with a greater fungal burden). To address if this γδ T cells from CD are impaired (as well being reduced in number) flow cytometry analysis is undertaken from cells isolated from the blood and stimulated with HMB-PP. Were these blood samples from the same individuals that provide intestinal samples? However, the presentation of these data is confusing. Flow plots of unstimulated vs HMB-PP plots are shown (Fig. 2E) , but it is unclear which of these are shown in graphs presented in Fig. 2F. Some markers are shown on plots but not quantified on the graphs (e.g. ICOS-L). Whereas the percentage of HLA-DR+ Vδ2+ is shown on graphs but no gate is shown on the plots to show how that data was achieved. This should be amended so that the presentation of the data on the flow plots and graphs are clearer. Furthermore, in Fig 2G I assume that CD4+ T cells expression of IL-22 was after culture with Vδ2+ γδ T cells? Can the authors show representative plots to confirm this staining of these populations?

3)The authors go onto show the diversity of C. albicans strains from colonic tissue (Fig. 3). It is unclear how many robust the data generated in A and B due to a lack of quantification. Have the authors undertaken any other approaches (e.g. sequencing) to confirm in an unbiased manner that C. albicans is the dominant st to other fungi in the tissue? Sequencing of the Candida isolates show little changes at the genomic level (Fig. 4) but that growing one of the stains (CA16) in hypoxic conditions were more filamentous compared to isolates from a healthy patient (Fig. 5). This is fascinating data, but could the authors give slightly more rationale on why they only focused on this one isolate? Did they test the other isolates they found and see different growth patterns in hypoxic conditions? Using pSILAC proteomics show a table of hits and present 4 example proteins identified. It would be of interest if the proteomics data could be more integrated with further analysis (e.g. Venn Diagrams) so the reader can ascertain how many proteins were altered/unaltered between the two strains in hypoxic vs normal growth conditions. The authors go on to show that CA16 activates elevated neutrophil and macrophages suggesting that CD isolates are more inflammatoty (Fig. 6). Yet, the reason for this isn't explored. Have the authors tested whether if this is due to CA16 having greater exposure of key fungal PAMPs like β-glucan (or that C. albicans from HC have lower exposure)?

4)The final figure utilises gut-on-a-chip to reveal that CA16 is more able to adhere and invade the intestinal barrier. The authors highlight this system does not have immune cells (e.g. γδ T cells) so they can't test if they help prevent this process. However, this is a very useful system that enables the authors to make some interesting observations about these isolates in a physiologically relevant setting. Have the authors attempted to alter the epithelium within these chips (e.g. to seed with epithelium from CD patients) to see how this might shape C. albicans behaviour and explain how CA16 originated? E.g. could CA47 from a HC become invasive if in a CD like gut environment?

# Author comment: Author response to Decision Letter 2

**DOI:** 10.1371/journal.pbio.3004080.r003

26 Aug 2026

Attachment Submitted filename: PLOSbio_Response to Reviewers.doc

# Peer review: Decision Letter 2

**DOI:** 10.1371/journal.pbio.3004080.r004

**Authors:** Melissa Vazquez Hernandez

21 Sep 2026

Dear Neil,

Thank you for your patience while we considered your revised manuscript "Fungal suppression by human intestinal γδ T-cells is disrupted in Crohn’s disease" for publication as a Research Article at PLOS Biology. This revised version of your manuscript has been evaluated by the PLOS Biology editors, the Academic Editor and the original reviewers.

Based on the reviews and on our Academic Editor's assessment of your revision, we are likely to accept this manuscript for publication, provided you satisfactorily address the following data and other policy-related requests.

1) We routinely suggest changes to titles to ensure maximum accessibility for a broad, non-specialist readership, and to ensure they reflect the contents of the paper. In this case, we would suggest a minor edit to the title, as follows. Please ensure you change both the manuscript file and the online submission system, as they need to match for final acceptance:

"Fungal suppression by bacterial metabolite-activated human intestinal γδ T cells is disrupted in Crohn’s disease"

2) Please add the weblink of the funding agencies in the Financial Disclosure statement in the manuscript details.

3) The Ethics statement needs to be a separate, independent (and the first) subheading in the Material & Methods section. Please include information about the form of consent (written/oral) given for research involving human participants. All research involving human participants must have been approved by the authors' Institutional Review Board (IRB) or an equivalent committee, and must have been conducted according to the principles expressed in the Declaration of Helsinki.

4) You may be aware of the PLOS Data Policy, which requires that all data be made available without restriction: http://journals.plos.org/plosbiology/s/data-availability. For more information, please also see this editorial: http://dx.doi.org/10.1371/journal.pbio.1001797

Please supply the numerical values either in the a supplementary file or as a permanent DOI’d deposition for the following figures:

Figure 1H, 2BFH, 3CDEF, 5CD, 6A-D, 7D, S3, S5AB, S6

NOTE: the numerical data provided should include all replicates AND the way in which the plotted mean and errors were derived (it should not present only the mean/average values).

5) Please cite the location of the data clearly in all relevant main and supplementary Figure legends, e.g. “The data underlying this Figure can be found in S1 Data” or “The data underlying this Figure can be found in https://doi.org/10.5281/zenodo.XXXXX”

6) Please provide the tree files for the phylogenetic trees in Figure S2. Please make sure all relevant figures have scale bars.

7) For figures containing FACS data (Figures 1G, 2AEG, S1), please provide the FCS files and a picture showing the successive plots and gates that were applied to the FCS files to generate the figure. We ask that you please deposit this data in an open depository like Zenodo and provide the accession number/URL of the deposition in the Data Availability Statement in the online submission form.

8) Please add a scale bar in all microscopy pictures

9) Please ensure that your Data Statement in the submission system accurately describes where your data can be found and is in final format, as it will be published as written there

10) Per journal policy, if you have generated any custom code during the course of this investigation, please make it available without restrictions. Please ensure that the code is sufficiently well documented and reusable, and that your Data Statement in the Editorial Manager submission system accurately describes where your code can be found. More information on our Code Policy, what and how to share can be found here: https://journals.plos.org/plosbiology/s/code-availability

Please note that we cannot accept sole deposition of code in GitHub, as this could be changed after publication. However, you can archive this version of your publicly available GitHub code to Zenodo. Once you do this, it will generate a DOI number, which you will need to provide in the Data Accessibility Statement (you are welcome to also provide the GitHub access information). See the process for doing this here: https://docs.github.com/en/repositories/archiving-a-github-repository/referencing-and-citing-content

As you address these items, please take this last chance to review your reference list to ensure that it is complete and correct. If you have cited papers that have been retracted, please include the rationale for doing so in the manuscript text, or remove these references and replace them with relevant current references. Any changes to the reference list should be mentioned in the cover letter that accompanies your revised manuscript.

In addition to these revisions, you may need to complete some formatting changes, which you will receive in a follow up email. A member of our team will be in touch with a set of requests shortly. If you do not receive a separate email within a few days, please assume that checks have been completed, and no additional changes are required.

We expect to receive your revised manuscript within two weeks.

To submit your revision, please go to https://www.editorialmanager.com/pbiology/ and log in as an Author. Click the link labelled 'Submissions Needing Revision' to find your submission record. Your revised submission must include the following:

- a cover letter that should detail your responses to any editorial requests, if applicable, and whether changes have been made to the reference list

- a Response to Reviewers file that provides a detailed response to the reviewers' comments (if applicable, if not applicable please do not delete your existing 'Response to Reviewers' file.)

- a track-changes file indicating any changes that you have made to the manuscript.

NOTE: If Supporting Information files are included with your article, note that these are not copyedited and will be published as they are submitted. Please ensure that these files are legible and of high quality (at least 300 dpi) in an easily accessible file format. For this reason, please be aware that any references listed in an SI file will not be indexed. For more information, see our Supporting Information guidelines:

https://journals.plos.org/plosbiology/s/supporting-information

*Published Peer Review History*

Please note that you may have the opportunity to make the peer review history publicly available. The record will include editor decision letters (with reviews) and your responses to reviewer comments. If eligible, we will contact you to opt in or out. Please see here for more details:

https://plos.org/published-peer-review-history/

*Press*

Should you, your institution's press office or the journal office choose to press release your paper, please ensure you have opted out of Early Article Posting on the submission form. We ask that you notify us as soon as possible if you or your institution is planning to press release the article.

*Protocols deposition*

To enhance the reproducibility of your results, we recommend that if applicable you deposit your laboratory protocols in protocols.io, where a protocol can be assigned its own identifier (DOI) such that it can be cited independently in the future. Additionally, PLOS ONE offers an option for publishing peer-reviewed Lab Protocol articles, which describe protocols hosted on protocols.io. Read more information on sharing protocols at https://plos.org/protocols?utm_medium=editorial-email&utm_source=authorletters&utm_campaign=protocols

Please do not hesitate to contact me should you have any questions.

Sincerely,

Melissa

Melissa Vazquez Hernandez, Ph.D.

Senior Editor

mvazquezhernandez@plos.org

PLOS Biology

------------------------------------------------------------------------

REVIEWERS' COMMENTS

------------------------------------------------------------------------

Reviewer #1:

All prior concerns have been adequately addressed. Congratulations to the authors on an excellent study.

------------------------------------------------------------------------

Reviewer #2 (Peter Cook):

I would like to thank the reviewer for addressing my queries and for providing further experimental data and rationale on their study design. I have no further comments.

# Author comment: Author response to Decision Letter 3

**DOI:** 10.1371/journal.pbio.3004080.r005

7 Oct 2026

Attachment Submitted filename: PLOSbio_Response_to_Reviewers_auresp_3.doc

# Editor report: Decision Letter 3

**DOI:** 10.1371/journal.pbio.3004080.r006

**Authors:** Melissa Vazquez Hernandez

13 Oct 2026

Dear Neil,

Thank you for the submission of your revised Research Article "Fungal suppression by bacterial metabolite-activated human intestinal γδ T cells is disrupted in Crohn’s disease" for publication in PLOS Biology. On behalf of my colleagues and the Academic Editor, Ken Cadwell, I am pleased to say that we can in principle accept your manuscript for publication, provided you address any remaining formatting and reporting issues. These will be detailed in an email you should receive within 2-3 business days from our colleagues in the journal operations team; no action is required from you until then. Please note that we will not be able to formally accept your manuscript and schedule it for publication until you have completed any requested changes.

Please take a minute to log into Editorial Manager at http://www.editorialmanager.com/pbiology/, click the "Update My Information" link at the top of the page, and update your user information to ensure an efficient production process.

PRESS

We frequently collaborate with press offices. If your institution or institutions have a press office, please notify them about your upcoming paper at this point, to enable them to help maximise its impact. If the press office is planning to promote your findings, we would be grateful if they could coordinate with biologypress@plos.org. If you have previously opted in to the early version process, we ask that you notify us immediately of any press plans so that we may opt out on your behalf.

We also ask that you take this opportunity to read our Embargo Policy regarding the discussion, promotion and media coverage of work that is yet to be published by PLOS. As your manuscript is not yet published, it is bound by the conditions of our Embargo Policy. Please be aware that this policy is in place both to ensure that any press coverage of your article is fully substantiated and to provide a direct link between such coverage and the published work. For full details of our Embargo Policy, please visit http://www.plos.org/about/media-inquiries/embargo-policy/.

Thank you again for choosing PLOS Biology for publication and supporting Open Access publishing. We look forward to publishing your study.

Sincerely,

Melissa

Melissa Vazquez Hernandez, Ph.D., Ph.D.

Senior Editor

PLOS Biology

mvazquezhernandez@plos.org
